# RNA‐seq profiling reveals different pathways between remodeled vessels and myocardium in hypertrophic cardiomyopathy

**DOI:** 10.1111/micc.12790

**Published:** 2022-10-14

**Authors:** Annalinda Pisano, Loredana Le Pera, Raffaella Carletti, Bruna Cerbelli, Maria G. Pignataro, Angelina Pernazza, Fabrizio Ferre, Maria Lombardi, Davide Lazzeroni, Iacopo Olivotto, Ornella E. Rimoldi, Chiara Foglieni, Paolo G. Camici, Giulia d'Amati

**Affiliations:** ^1^ Department of Radiological, Oncological and Pathological Sciences Sapienza University of Rome Rome Italy; ^2^ Italian National Institute of Health (ISS), Core Facilities Rome Italy; ^3^ National Research Council (IBIOM‐CNR) Institute of Biomembranes, Bioenergetics and Molecular Biotechnologies Bari Italy; ^4^ Department of Translational and Precision Medicine Sapienza University of Rome Rome Italy; ^5^ Department of Medico‐Surgical Sciences and Biotechnologies Sapienza University of Rome Latina Italy; ^6^ Department of Chemistry and Drug Technologies Sapienza University of Rome Rome Italy; ^7^ Department of Pharmacy and Biotechnology (FABIT) University of Bologna Bologna Italy; ^8^ Cardiovascular Research Center IRCCS San Raffaele Scientific Institute Milan Italy; ^9^ Cardiomyopathy Unit Careggi University Hospital Florence Italy; ^10^ National Research Council (IBFM‐CNR) Institute of Molecular Bioimaging and Physiology Milan Italy; ^11^ Faculty of Medicine and Surgery Vita‐Salute University Milan Italy

**Keywords:** coronary microvascular remodeling, hypertrophic cardiomyopathy, pathway enrichment analyses, remodeled arterioles dissected

## Abstract

**Objective:**

Coronary microvascular dysfunction (CMD) is a key pathophysiological feature of hypertrophic cardiomyopathy (HCM), contributing to myocardial ischemia and representing a critical determinant of patients' adverse outcome. The molecular mechanisms underlying the morphological and functional changes of CMD are still unknown. Aim of this study was to obtain insights on the molecular pathways associated with microvessel remodeling in HCM.

**Methods:**

Interventricular septum myectomies from patients with obstructive HCM (*n* = 20) and donors' hearts (CTRL, discarded for technical reasons, *n* = 7) were collected. Remodeled intramyocardial arterioles and cardiomyocytes were microdissected by laser capture and next‐generation sequencing was used to delineate the transcriptome profile.

**Results:**

We identified 720 exclusive differentially expressed genes (DEGs) in cardiomyocytes and 1315 exclusive DEGs in remodeled arterioles of HCM. Performing gene ontology and pathway enrichment analyses, we identified selectively altered pathways between remodeled arterioles and cardiomyocytes in HCM patients and controls.

**Conclusions:**

We demonstrate the existence of distinctive pathways between remodeled arterioles and cardiomyocytes in HCM patients and controls at the transcriptome level.

**Abbreviations**
BP: biological processes
CC: cellular compartment
CMD: coronary microvascular dysfunction
DEGs: differentially expressed genes
FDR: false discovery rate
GO: gene ontology
HCM: hypertrophic cardiomyopathy
HE: hematoxylin‐eosin
LA: lumen area
LCM: laser capture microdissection
LVH: left ventricular hypertrophy
MF: molecular functions
NGS: next‐generation sequencing
RIN: RNA integrity number
SEM: standard error of the mean
VA: vessel area

## 1 INTRODUCTION

Hypertrophic cardiomyopathy (HCM) is the most common genetic heart disease, with a prevalence of 1:500 in the general population^1^ and is mainly caused by single‐gene mutations in genes encoding proteins of the sarcomere.^2^, ^3^, ^4^, ^5^ The mutations lead to structurally and functionally altered proteins,^3^ generating a cascade of secondary defects in cardiomyocyte energetics, contractility, and structure.^6^

Macroscopically HCM demonstrates marked left ventricular hypertrophy (LVH), often asymmetric,^7^, ^8^, ^9^ and ventricular dysfunction. Microscopically, it is characterized by cardiomyocyte hypertrophy and heterogeneously distributed spatial disarray, interstitial fibrosis and adverse remodeling of intramural coronary arterioles (i.e., vessel wall thickening with hypertrophy of smooth muscle cells and increased collagen deposition in the tunica media with variable degrees of intimal thickening) with local ischemia.^10^, ^11^, ^12^

Coronary microvascular dysfunction (CMD) represents a key pathophysiological mechanism in HCM, contributing to myocardial ischemia and replacement fibrosis,^13^, ^14^, ^15^, ^16^ pointing to CMD as a critical determinant of adverse outcome in HCM. Whether CMD is part of the same gene deregulation associated to myocardial alteration is unknown.

In the last decades, the development of laser capture microdissection (LCM) allowed high‐resolution isolation of selected cells/tissue portions from tissue sections, preserving the molecular composition for omics analyses.^17^, ^18^, ^19^, ^20^, ^21^, ^22^, ^23^, ^24^, ^25^, ^26^ Recently, the coupling of LCM with next‐generation sequencing (NGS) has been proposed as powerful strategy to investigate and compare the transcriptome profiles of different components of the same samples, but low quantities of starting RNA can be a severe hindrance, especially for RNA‐sequencing studies. Multiple protocols have been developed for transcriptome profiling from very low‐quantity RNA inputs^27^, ^28^, ^29^ and recent advances in RNA‐sequencing technology enable sequencing analysis with limited amounts of RNA obtained from selected areas of interest.^30^, ^31^, ^32^

In the current study, we applied the combined approach of LCM and full‐length mRNA‐sequencing to compare the transcriptome profiling of remodeled arterioles and cardiomyocytes from interventricular septal tissue of HCM patients vs. controls (CTRL, donor hearts). Differentially expressed genes (DEGs) were identified and analyzed by comparative functional enrichment to obtain insights on the pathways putatively associated with HCM and related either to cardiomyocyte alterations or CMD.

## 2 MATERIALS AND METHODS

### 2.1 Sample collection

The work described has been carried out in accordance with The Code of Ethics of the World Medical Association (Declaration of Helsinki) and conformed to Sapienza University of Rome Ethical Committee protocols.

Myocardial samples were obtained from patients with obstructive HCM, diagnosed according to current guidelines,^7^ undergoing septal myectomy procedures at Careggi University Hospital, Florence (*n* = 4) and San Raffaele Hospital, Milan (*n* = 16). All patients gave informed consent for the procedure.

Control myocardial samples (CTRL, *n* = 7) from the same site of the septal myectomy procedure (subaortic septum) were collected at Sapienza University Hospital in Rome from donors' hearts discarded from transplantation because of noncardiac technical reasons (e.g., suitable recipient unavailability).

Samples were harvested immediately after surgery and cut into 2‐mm thick slices perpendicularly to the endocardium. Part of each specimen was embedded in KilliK (O.C.T. BioOptica) and snap‐frozen in nitrogen‐cooled isopentane for in situ gene‐expression studies. The remaining tissue was fixed in 10% phosphate‐buffered formalin and embedded in paraffin for histological and histomorphometric analyses.

### 2.2 Histological and histomorphometric analysis

Eight‐μm‐thick sections were obtained from each paraffin block, de‐paraffinized, rehydrated and stained with hematoxylin‐eosin (HE) and Azan Mallory stain for light microscopy. For the purpose of the study, small vessels were defined as intramural arterioles with a diameter <100 μm.^13^ For each case, the small vessel medial hypertrophy and perivascular fibrosis, as well as the presence of myocardial hypertrophy, myofiber disarray, interstitial fibrosis, and myocardial microscarring were recorded. High‐resolution images of intramural arterioles were acquired at 20× magnification with a digital camera (Olympus). Images were stored as TIF files and were analyzed by a dedicated software (ImageJ 1.47v, Wayne Rasband National Institutes of Health). Lumen area (LA) and vessel area (VA) were manually measured. The following parameters were then derived: Medial area (VA‐LA); Lumen area to vessel area ratio (LA/VA); Lumen diameter [√(LA∕3.14)] × 2 and Vessel diameter [√(V∕3.14)] × 2 as previously published.^33^

### 2.3 Statistical analysis

For the histomorphometric analysis, all data are reported as mean ± standard error of the mean (SEM). For direct comparison between HCM patients and controls, unpaired Student's *t* test was used. Significance was considered at *p* < .05. Numerical estimates were obtained with the GraphPad Prism 7 version (GraphPad Inc).

### 2.4 LCM procedure

Frozen sections from the myectomy specimens with a thickness of 10 μm were mounted onto PEN‐membrane glass slides (Leica Microsystems) and stained with HE. Between 100–150 remodeled intramyocardial arterioles were microdissected by laser capture with the Leica LMD 7000 (Leica Microsystems). For each sample, similar amounts of dissected cardiomyocytes were also obtained (Figure S1).

### 2.5 RNA preparation

Total RNA from microdissected samples was isolated by miRNeasy Micro Kit (Qiagen) specific for purifying total RNA from small amounts of tissue. The concentration and purity of total RNA were determined using Agilent RNA 6000 Pico Assay (Agilent 2100 Bioanalyzer). Only the samples presenting RNA Integrity Number (RIN) ≥ 7 were selected for the further next‐generation sequencing study.

### 2.6 RNA‐sequencing

RNA was converted into RNA‐seq libraries with the Clontech Smarter kit (specifically designed for RNA‐Seq applications involving laser‐captured samples)^34^ and sequenced on an Illumina Nextseq 500 sequencer with a HighOutput flow cell, 1 × 75 nt, single‐end reads.

### 2.7 RNA‐seq data analysis

#### 2.7.1 Pre‐processing

Quality control for 75‐base single‐end reads of each sample was performed by using the FastQC tool,^35^ which can examine sequence quality, GC content, presence of adaptors, over‐represented k‐mers, and read duplication. The Trimmomatic software (v. 0.36)^36^ was used to discard low‐quality reads (average quality <28), eliminate poor‐quality bases from their 3′ end, and trim adaptor sequences. Only reads longer than 35 bases were retained and mapped on the human reference genome (GRCh38) by using HISAT2 aligner (v2.1.0)^37^ with default parameter values.

#### 2.7.2 Differential expression analysis (HCM versus CTRL) in myocardium and vessel tissue

Read counts for each human gene were estimated by using StringTie software (v2.1.1) with the human transcriptome from Ensembl (release 98) as reference annotation, followed by running the prepDE.py Python script to generate the count matrix (as provided and suggested in the StringTie protocol).^38^ Mitochondrial genes were excluded from the analysis to avoid the introduction of significant biases in the differential expression analysis due to their high expression levels in the myocardium.^39^ Principal Component Analysis (PCA) and correlation coefficient analysis were performed to examine gene‐expression level of HCM and CTRL samples both in cardiomyocytes and vessels, assessing similarities and differences between groups. Normalization and differential expression test were performed using DESeq2 R‐package (v1.26).^40^ For each comparison, only genes with more than 1 count per million (cpm) in a minimum number of samples (the size of the group with the lowest number of samples under analysis) were retained. Multiple‐testing correction to control the false discovery rate (FDR) was performed by applying Benjamini–Hochberg method.^41^ Only the genes with an adjusted *p* < .01 were marked as differentially expressed genes (DEGs) (significantly up‐ and down‐regulated). As the biological importance of a given change in expression level is unknown, no fold‐change cutoff was applied. Genes that were differentially expressed (adjusted *p* < .01) between HCM versus CTRL in myocardium, but not in vessels (adjusted *p* > .01), were marked as exclusive myo‐DEGs, and vice versa for exclusive vessel‐DEGs. Gene overlaps were calculated using the InteractiVenn software.^42^

### 2.8 Functional enrichment analysis

A functional enrichment analysis was performed on each set of differentially expressed (protein‐coding) genes between HCM and CTRL, both in myocardium and arterioles, keeping the up‐ and down‐regulated groups. Over‐represented biological processes, functions, or pathways (terms which have more DEGs than expected by chance) were identified by using DAVID web‐server^43^ with the entire human proteome as reference and querying the following functional categories: Gene Ontology (GO) terms^44^ related to Biological Processes (BP), Molecular Functions (MF), and Cellular Compartment (CC); protein families as classified by InterPro database^45^; pathways collected in KEGG^46^ and Reactome^47^ databases; UniProt^48^ protein annotations, and putative molecular interactors as annotated in IntAct database.^49^ Only biological categories with Benjamini–Hochberg corrected *p*‐value^40^ (adjusted *p*‐value) ≤5 × 10^−2^ were considered as statistically enriched. Results for each investigated group (up‐ and down‐regulated protein‐coding genes, both in myocardium and vessel) are shown as heatmaps, with the color scale representing the adjusted *p*‐values, created using the gplots R‐package (https://CRAN.R‐project.org/package=gplots).

## 3 RESULTS

### 3.1 Study population

Twenty patients with a diagnosis of obstructive HCM according to current guidelines^7^ were enrolled. Baseline clinical and echocardiographic data are summarized in Table 1. Seven CTRL biopsies were collected. Due to privacy law, only data regarding age and sex of donors were available. The control group consisted of 4 males and 3 females, with a mean age of 55 ± 8 years.

**TABLE 1:** Baseline characteristics and echocardiographic data of HCM patients

Patients	*N* = 20
Demographic data	
Age (years), M (SD)	59 (8)
Male gender, N (%)	14 (70)
BMI (kg/m^2^), M (SD)	27 (5)
Clinical data	
Positive genetic screening, *N* (%)	7 (35)
VUS, *N* (%)	3 (15)
Family history of HCM, *N* (%)	7 (35)
NYHA class ≥ III, *N* (%)	8 (40)
Angina, *N* (%)	2 (10)
Syncope, *N* (%)	4 (20)
NSVT, *N* (%)	1 (5)
Medical therapy	
Beta‐blockers, *N* (%)	18 (90)
Antiarrhythmic drugs, *N* (%)	4 (20)
Diuretics, *N* (%)	12 (60)
RAAS‐i, *N* (%)	12 (60)
CCB, *N* (%)	3 (15)
Echocardiographic data	
IVS thickness (mm), M (SD)	22 (5)
LV‐EDV (ml), M (SD)	141 (62)
LV‐EF (%), M (SD)	67 (9)
Moderate‐to‐severe mitral regurgitation, %	85
SAM‐related LVOT‐max gradient at rest (mm Hg), M (SD)	68 (35)

Abbreviations:aldosterone system inhibitors; angiotensin‐ BMI, body mass index; CCB, calcium channel blockers; EDV, end diastolic volume; EF, ejection fraction; IVS, interventricular septum; LV, left ventricular; LVOT, left ventricular outflow tract; M, mean; Max, maximum; *N*, number; NSVT, non‐sustained ventricular tachycardia; NYHA class, New York Heart Association class; RAAS‐i, renin‐ SD, standard deviation; VUS, variants of uncertain significance.

### 3.2 Histologic and morphometric features

Histologic evaluation of myectomy samples was in keeping with the clinical diagnosis of HCM, showing myocyte hypertrophy and areas of myofiber disarray, characterized by bundles of myocytes crossing each other with a herringbone pattern. Microscopic examination also revealed the presence of both interstitial and replacement fibrosis, the latter frequently surrounding remodeled coronary arterioles, with medial wall thickening, mainly due to smooth muscle hypertrophy and increased collagen deposition and variable intimal thickening. Histologic analysis of control samples showed a normal myocardial and vessel structure (Figure S2).

Morphometric analysis showed a significant increase in microvascular medial area in HCM samples as compared to CTRL, (9578.59 ± 1295.76 μm^2^ vs 3752.03 ± 536.7 μm^2^, *p* = .0001) paralleled by a decrease of the lumen‐ to vessel area ratio (0.10 ± 0.01 vs 0.24 ± 0.02 in HCM patients and controls, *p* = .0001).

### 3.3 Detection of differentially expressed genes in HCM versus CTRL, common and specific to myocardium and vessel tissues

NGS was used to delineate the transcriptome profile of cardiomyocytes and arterioles in HCM and CTRL samples. For these experiments, we dissected arterioles from 20 HCM hearts and 6 CTRL, and cardiomyocytes from 10 HCM and 5 CTRL, selected for RNA quality (RIN ≥ 7).

Reads univocally mapped on the human reference genome (>70% of the sequenced reads) were used to estimate the gene‐expression values in all samples (Figures S3 and S4). Exploratory gene‐expression PCA plots (with respect to the first two components) visualized the distribution of the samples (Figures S5 and S6), showing an overall separation between HCM and CTRL. The higher dispersion of CTRL revealed more heterogeneous expression with respect to clustered HCM points, indicative of inherently greater variability in the healthy CTRL than in HCM patients.

**FIGURE 1:**
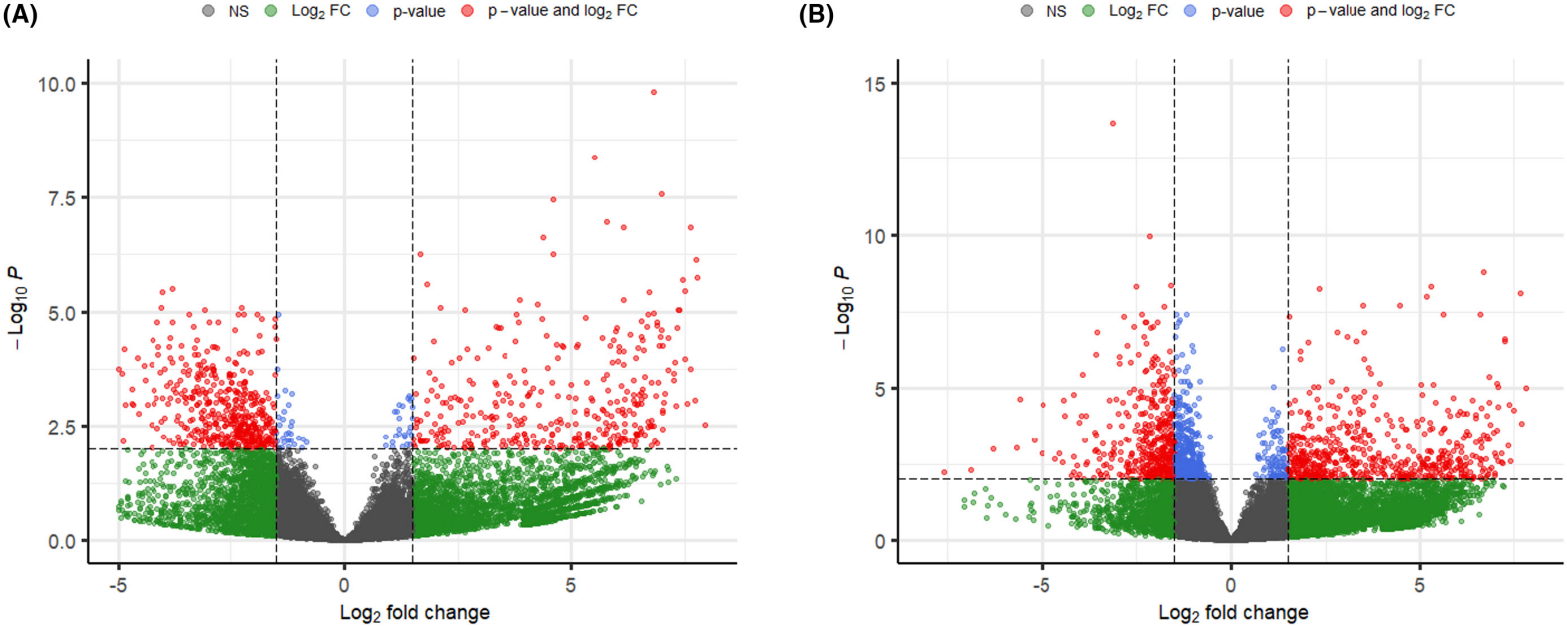
“Volcano plot” showing statistical significance (adjusted *p*‐values, *p*) versus magnitude of change (fold change, FC) of gene‐expression data between HCM and CTRL in cardiomyocytes (A) and arterioles (B). The most up‐regulated genes (log_2_FC > 2) are towards the right, the most down‐regulated genes (log_2_FC < −2) are towards the left, with the statistically significant data (−log_10_P > 2) highlighted in red, while the not significant ones in green. In blue, differential expression data with significant adjusted *p*‐values but small fold changes are reported; in gray, not significant (NS) differential expression data.

The Volcano Plots (Figure 1) showed a balanced (symmetrical) distribution of data, revealing no alterations in the results due to bias or artifacts between HCM and CTRL.

Our technique allowed the detection of a high number of genes differentially expressed between HCM and normal heart, both in cardiomyocytes and in remodeled arterioles. In fact, a total of 890 differentially expressed genes (DEGs) were detected in cardiomyocytes (387 DEGs up‐ and 503 DEGs down‐regulated) and 1485 DEGs were identified in the remodeled arterioles (675 up‐regulated and 810 down‐regulated, Tables S1 and S2).

**FIGURE 2:**
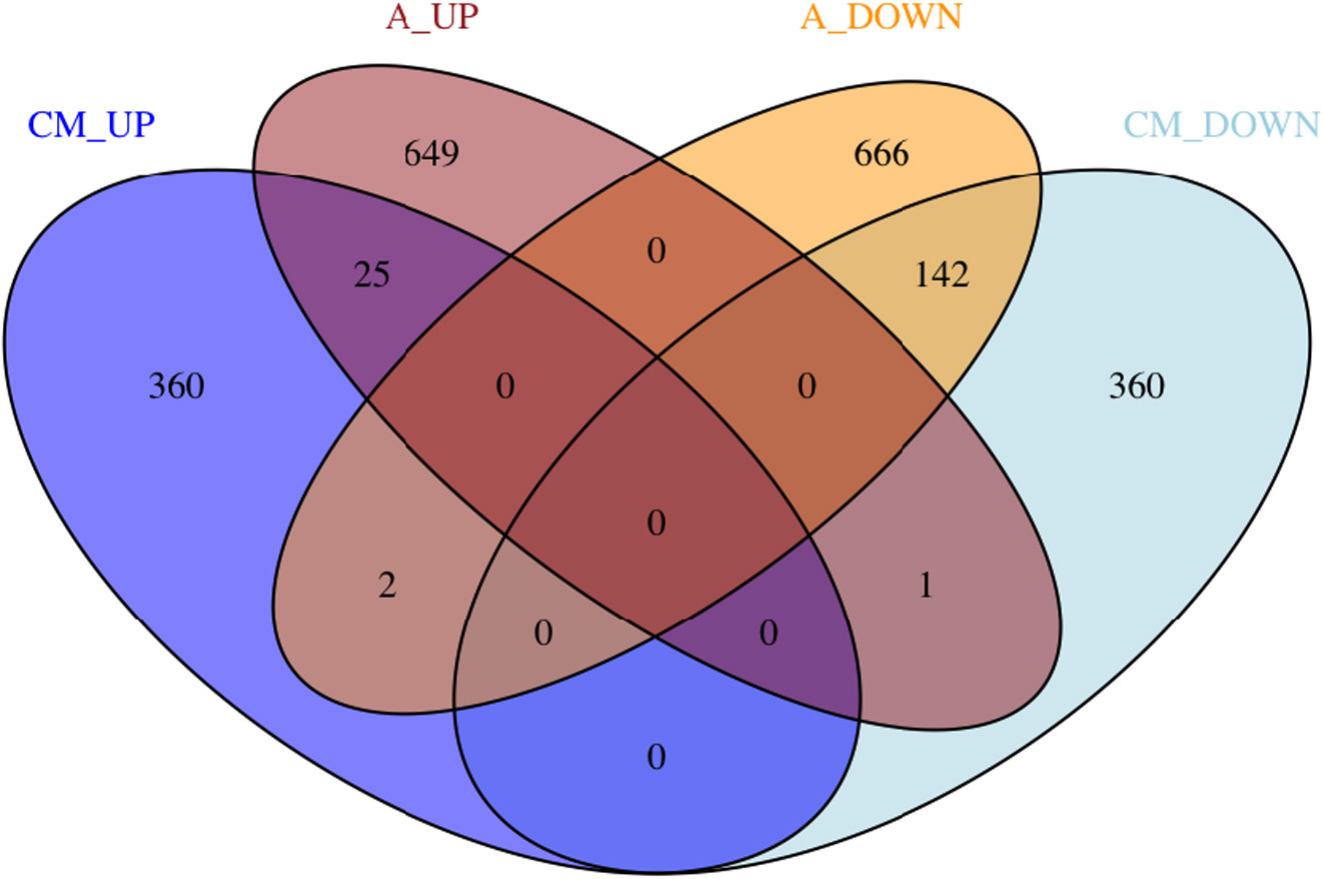
Venn diagram of differentially expressed genes (DEGs) identified using DESeq2. The panel shows the overlap between up‐ and down‐regulated genes in cardiomyocytes (CM_UP, CM_DOWN) and vessels arterioles (A_UP, A_DOWN), highlighting the number of genes deregulated in both tissues (in common) or in a specific tissue (exclusive DEGs). CM_DOWN, cardiomyocytes_DOWN; CM_UP, cardiomyocytes_UP; A_DOWN, arterioles_DOWN; A_UP: arterioles_UP.

Among the differentially expressed genes, 170 were altered both in HCM cardiomyocytes and arterioles. Of those, 25 were parallel up‐regulated and 142 down‐regulated (Figure 2). Conversely, two genes, the C‐type lectin domain‐containing 16A (CLEC16A) and Myeloid/lymphoid or mixed‐lineage leukemia; translocated to, 11 (MLLT11, an inducer of bad‐mediated intrinsic apoptosis), were up‐regulated in cardiomyocytes and down‐regulated in arterioles. Only one gene, the IQ domain‐containing protein N (IQCN), was down‐regulated in cardiomyocytes and up‐regulated in arterioles.

Interestingly, we were able to identify “exclusive DEGs” either for cardiomyocytes or arterioles, that is, DEGs between HCM and control in cardiomyocyte but not in remodeled arterioles samples and vice versa. Moreover, we found that the vast majority of DEGs are indeed exclusive: 360 (out of 387) and 360 (out of 503) up‐ and down‐regulated, respectively, in cardiomyocytes, and 649 (out of 675) and 666 (out of 810) up‐ and down‐regulated, respectively, in arterioles (Figure 2, Table 2 and Tables S3 and S4).

**TABLE 2:** Top 10 exclusive DEGs down‐ an up‐ regulated in dissected cardiomyocytes and arterioles

Gene name	Description	*p*‐value	Gene name	Description	*p*‐value
Cardiomyocytes_DOWN			Cardiomyocytes_UP		
IGKV3‐20	Immunoglobulin kappa variable 3–20	3.35E‐13	ABCD2	ATP binding cassette subfamily D member 2	1.60E‐10
MYH11	Myosin heavy chain 11	2.70E‐08	ICA1L	Islet cell autoantigen 1 like	4.16E‐09
ACTA2	Actin alpha 2, smooth muscle	2.70E‐08	STRIT1	Small transmembrane regulator of ion transport 1	2.70E‐08
TCEA2	Transcription elongation factor A2	3.24E‐06	SLC25A40	Solute carrier family 25 member 40	3.58E‐08
USF2	Upstream transcription factor 2, c‐fos interactin	8.38E‐06	HSD11B1	Hydroxysteroid 11‐beta dehydrogenase 1	3.69E‐08
RRAGC	Ras‐related GTP binding C	8.38E‐06	ASB5	Ankyrin repeat and SOCS box containing 5	1.43E‐07
CALD1	Caldesmon 1	9.00E‐06	TTC30A	Tetratricopeptide repeat domain 30A	2.38E‐07
MRPL38	Mitochondrial ribosomal protein L38	1.13E‐05	SIX4	SIX homeobox 4	3.05E‐07
SGCA	Sarcoglycan alpha	1.13E‐05	RNF181	Ring finger protein 181	5.50E‐07
REEP3	Receptor expression‐enhancing Protein 3	1.43E‐05	TMEM177	Transmembrane protein 177	5.50E‐07
Arterioles _DOWN			Arterioles _UP		
WAC‐AS1	WAC antisense RNA 1 (head to head)	2.08E‐14	TRIM36	Tripartite motif containing 36	1.55E‐09
EHMT1	Euchromatic histone lysine methyltransferase 1	1.10E‐10	MUSTN1	Musculoskeletal, embryonic nuclear protein 1	5.74E‐09
GNB2	G protein subunit beta 2	2.14E‐08	TOB1‐AS1	TOB1 antisense RNA 1	7.91E‐09
IL17RA	Interleukin 17 receptor A	3.72E‐08	ZNF227	Zinc finger protein 227	9.99E‐09
MTDH	Metadherin	3.72E‐08	AVPI1	Arginine vasopressin induced 1	1.96E‐08
CAST	Calpastatin	5.97E‐08	C12orf66	KICSTOR subunit 2	1.96E‐08
FKBP8	FKBP prolyl isomerase 8	6.68E‐08	IGSF1	Immunoglobulin superfamily member 1	3.72E‐08
NES	Nestin	6.77E‐08	TMSB4X	Thymosin beta 4 X‐linked	4.46E‐08
AGPAT2	1‐Acylglycerol‐3‐Phosphate O‐Acyltransferase 2	6.95E‐08	ZNF79	Zinc finger protein 79	6.08E‐08
GPATCH8	G‐patch domain‐containing 8	9.56E‐08	CHURC1‐FNTB	CHURC1‐FNTB Readthrough	9.56E‐08

### 3.4 Comparative functional enrichment analyses identify tissue‐specific pathways potentially altered in CMD

To detect DEGs potentially involved in HCM pathogenesis, we performed Gene Ontology and Pathway enrichment analyses, two fundamental investigations exploring expression data. More specifically, comparative functional analyses were performed across up‐ and down‐regulated DEGs in HCM cardiomyocytes and arterioles by using Gene Ontology terms (BP, CC, and MF), KEGG and Reactome Pathways, InterPro domains, UniProt protein annotations, and the IntAct molecular interaction annotations (Figures S7–S10). Enriched biological processes and pathways included terms such as proteasome (GO0005839, hsa03050), apoptosis (R‐HSA‐109581), PIP3 activates AKT signaling (R‐HSA‐1257604) and MAPK family signaling cascades (R‐HSA‐5683057) (Figures S7 and S8). The enrichment results highlighted that the most down‐regulated DEGs, both in cardiomyocytes and coronary arterioles, are those encoding phosphoproteins (Figure S9).

**FIGURE 3:**
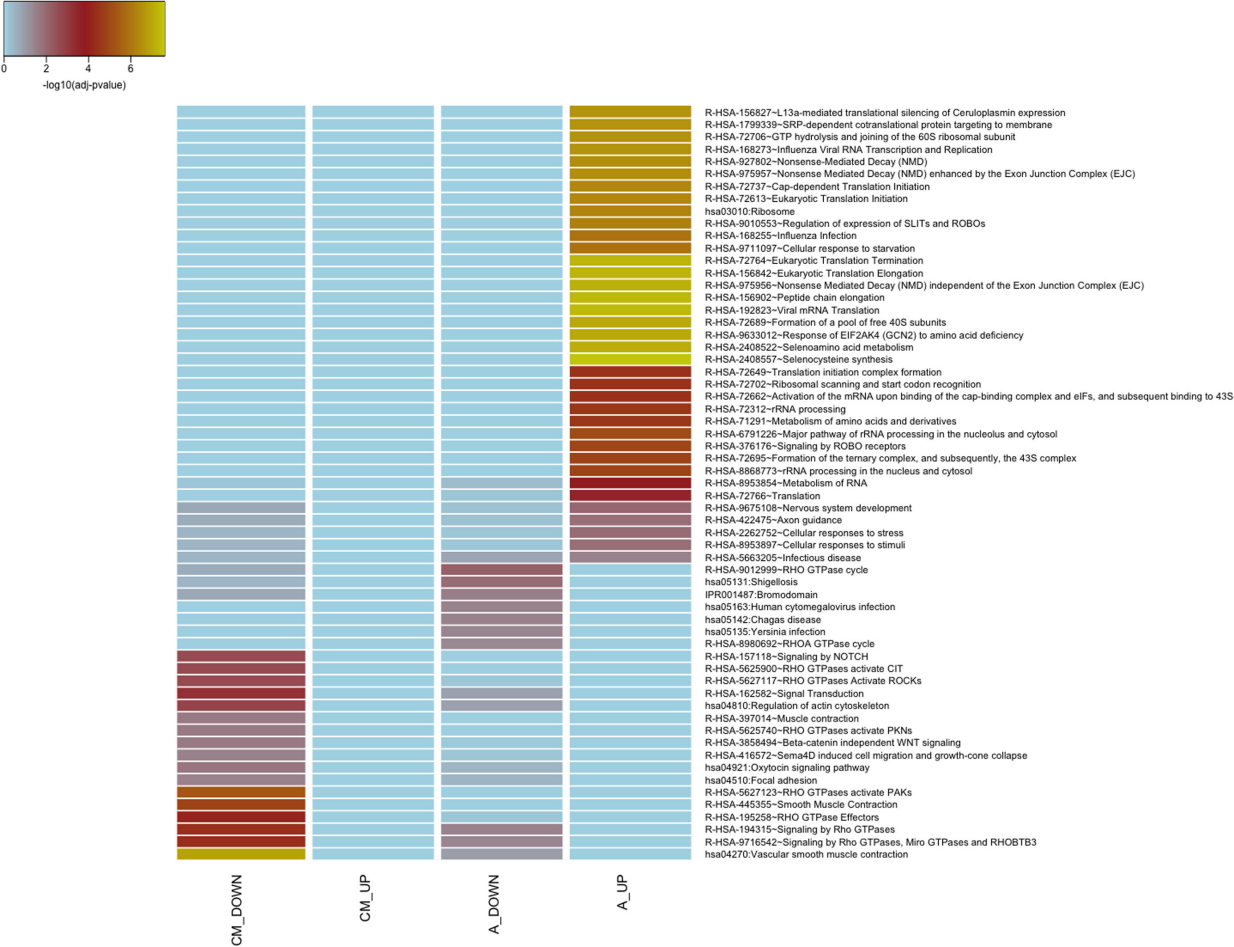
Comparison of KEGG and Reactome Pathways enriched in the list of “exclusive DEGs”. Terms statistically enriched in at least one of the four groups of “exclusive DEGs”: cardiomyocytes_DOWN (CM_DOWN), cardiomyocytes_UP (CM_UP), arterioles_DOWN (A_DOWN), arterioles_UP (A_UP) are reported as a heatmap, with adjusted *p*‐values plotted in blue‐yellow scale color, where yellow indicates higher significant results.

Among these biological pathways and functional categories, several were enriched only by “exclusive DEGs,” and more likely altered in a tissue‐specific way. In fact, the KEGG and Reactome pathways analysis (Figure 3) on “exclusive DEGs” demonstrated an evident separation between the contributions of cardiomyocytes and arterioles in HCM and CTRLs, confirmed by corresponding GO and UniProt terms (Figures S11 and S12). Several pathways (Figure 3) related to the “translation process,” such as “peptide chain elongation,” “ribosome,” “Nonsense Mediated Decay independent of/enhanced by the exon junction complex” were enriched by genes up‐regulated in arterioles, while “regulation of actin cytoskeleton” and “focal adhesion” pathways, and pathways related to muscle contraction, such as “vascular smooth muscle contraction,” or to signal transduction, such as “RHO GTPases activate PAKs/PKNs/ROCKs,” were enriched by genes down‐regulated in cardiomyocytes.

**FIGURE 4:**
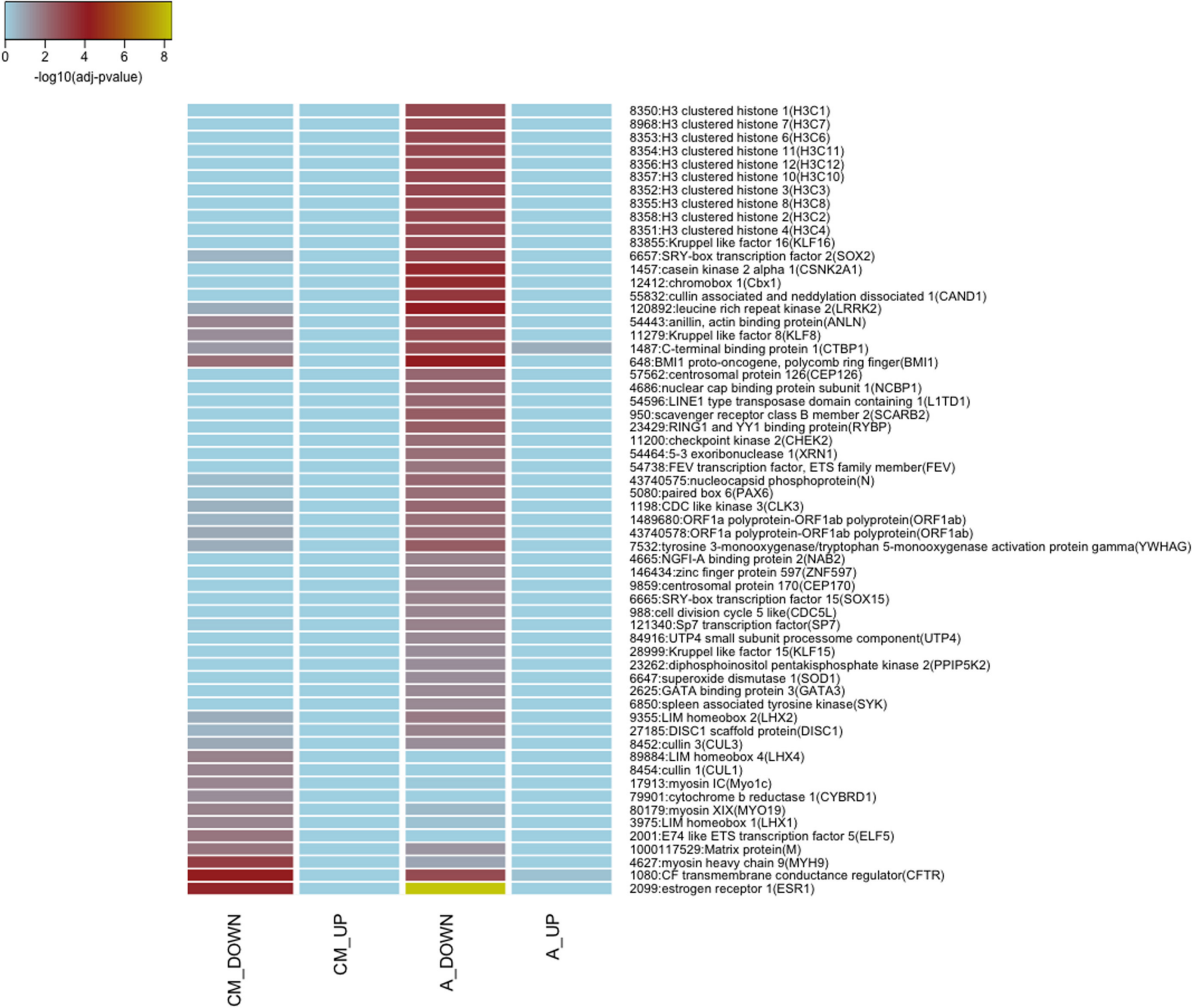
Comparison of Interactors (IntAct database) enriched in the list of “exclusive DEGs”. Terms statistically enriched in at least one of the four groups of “exclusive DEGs”: cardiomyocytes_DOWN (CM_DOWN), cardiomyocytes_UP (CM_UP), arterioles_DOWN (A_DOWN), arterioles_UP (A_UP) are reported as a heatmap, with adjusted *p*‐values plotted in blue‐yellow scale color, where yellow indicates higher significant results.

Notably, from the data stored in the IntAct database (Figure 4), among proteins with interactors enriched in genes exclusively down‐regulated in cardiomyocytes, we found myosin IC (Myo1c), myosin XIX (MYO19), and myosin heavy chain 9 (MYH9); myosins are actin‐based motor proteins that are required for multiple functions ranging from cytokinesis to muscle contraction.^50^

Among the down‐regulated genes in arterioles, we found an enrichment of interactors for two member of SOX family, SRY‐box transcription factor 2 and 15 (SOX2 and SOX15). The members of the SOX family of transcription factors widely expressed in development and participate in vasculogenesis and remodeling.^51^

## 4 DISCUSSION

Hypertrophic cardiomyopathy is the most common genetic cardiomyopathy with a phenotype characterized by massive left ventricular hypertrophy (LVH), myocyte disarray, interstitial fibrosis, and coronary microvascular disease. The latter includes abnormal wall thickening of intramural coronary arterioles with lumen reduction which correlates with the decrease in maximum myocardial blood flow and coronary flow reserve.^10^

Consistent evidence points to coronary microvascular dysfunction as a critical determinant of clinical progression and adverse outcome in HCM.^14^, ^16^ However, little is known regarding the pathogenic mechanisms underlying this condition.

In recent years, whole transcriptome investigations by performing focused RNA‐seq experiments and/or analyzing related datasets available in specialized online repositories revealed altered gene‐expression profiles in HCM. Reported gene‐enriched pathways involve immune modulation, signal transduction, hemostasis, metabolism, muscle contraction, inflammation, and fibrosis (TGF‐β pathways).^52^, ^53^, ^54^, ^55^

Results published so far are based on the analysis of whole myocardial samples, including cardiomyocytes, vessels, fibroblasts, and interstitial tissue, and do not provide information on the possible existence of expression profiles selectively related to coronary microvascular dysfunction.

In the last decade, the combination of LCM and RNA‐seq has been proven to be a useful tool to investigate cellular pathways underlying specific diseases and to identify potential therapeutic targets.^56^, ^57^ Compared with other cell isolation techniques, LCM can precisely target and capture the cells of interest for a wide range of downstream assays.^25^, ^26^, ^58^, ^59^

To gain insight into the molecular mechanisms of CMD in HCM, we isolated remodeled arterioles and cardiomyocytes by LCM from frozen myectomy samples and investigated their respective transcriptome profiles by RNA‐seq.

By applying this technique, we identified a total of 1485 differentially expressed genes (DEGs) in remodeled arterioles of HCM, of which 675 were over‐expressed and 810 under‐expressed. A lower number of DEGs (890) was detected in cardiomyocytes, of which 387 were up‐regulated and 503 were down‐regulated.

Interestingly, over 80% of the genes differentially expressed were exclusive of arterioles or cardiomyocytes (“exclusive DEGs” ie genes differentially expressed only in cardiomyocytes or in remodeled arterioles as compared to controls).

Pathway enrichment analysis of these “exclusive DEGs” recognized pathways specifically related to hypertrophic cardiomyocytes. We identified a down regulation of pathways correlated to muscle contraction, such as “vascular smooth muscle contraction” and “smooth muscle contraction” and signaling transduction, such as “RHO GTPases activate PAKs/PKNs/ROCKs.” Rho GTPases are key regulators of different actomyosin‐based cellular processes such as cell adhesion, cytokinesis, and contraction. The small GTP‐binding proteins of the Ras family, such as RhoA, stabilize the actin cytoskeleton and promote the formation of focal adhesions.^60^, ^61^, ^62^ Accordingly, we also observed a down regulation of pathways related to “regulation of actin cytoskeleton” and “focal adhesion.”

In contrast, remodeled arteries isolated by LCM showed selective alterations of pathways related to both the translation process (such as “peptide chain elongation,” and “ribosome”) and RNA quality control (“Nonsense Mediated Decay independent of/enhanced by the exon junction complex”).

Interestingly, these pathways appear to be specific of remodeled arterioles in HCM since they have not been previously described in vascular remodeling associated with other cardiovascular diseases, including atherosclerosis and hypertension. Several studies, in fact, have highlighted the role of specific pathways related to immune/inflammatory process or to signaling transduction, such as Rho/ROCK pathways, Hippo/YAP signaling, and TGF‐β pathway in vascular dysfunction associated with this condition.^63^, ^64^, ^65^, ^66^

To the best of our knowledge, this is the first study that demonstrates the existence of distinctive pathways modifications between remodeled arterioles and cardiomyocytes in HCM patients and controls at the transcriptome level. The results obtained on isolated cardiomyocytes are overlapping, at least partially, with previous studies analyzing HCM myocardial homogenates.^52^, ^53^, ^54^, ^55^ This might reflect the relative abundance of cardiomyocytes in the myocardial samples. Conversely, the association of LCM and RNA‐seq allowed the detection of previously undescribed altered pathways, exclusive of remodeled arterioles and selective for HCM. This finding emphasizes the usefulness of this approach to analyze the molecular mechanisms underlying microvascular dysfunction and, possibly, to identify putative therapeutic targets.

### 4.1 Limitation of the study

The present study is a preliminary analysis, which requires validation by RT‐PCR or Western blot analyses that have not yet been performed since all tissue samples collected for this study were used for NGS experiments. However, our main aim was to look for pathways specifically related to microvessel remodeling in HCM.

## 5 PERSPECTIVES

Our results highlight the usefulness of LCM/RNA‐seq to identify specific molecular pathways related to CMD in HCM. Functional validation is needed to identify putative target genes amenable to future therapeutic approaches. To this purpose, we are collecting additional samples to extend our preliminary results and to perform a functional validation of the identified genes by RT‐PCR and Western blot analysis.

## AUTHOR CONTRIBUTIONS

AP, GdA, and PGC designed and supervised the study; AP, RC, and MGP performed the experiments; AP, LLP, FF, and GdA analyzed the data; AP, LLP, and GdA wrote the manuscript; LLP, FF, BC, and APe prepared figures; ML, DL, and IO collected clinical data; CF, IO, OER, and PGC critically revised the draft.

## FUNDING INFORMATION

This work was supported by the Ministero della Salute‐Ricerca finalizzata 2011–2012 (P.G.C., NET‐2011‐02347173).

## CONFLICT OF INTEREST

The authors declare that they have no conflict of interest.

## Supporting information


Table S1

Table S2

Table S3

Table S4

Figures S1–S12

## Data Availability

We submitted the raw files (fastq) to the European Nucleotide Archive (ENA) under the project accession: PRJEB52994.
