# Supplementary material for: RNA‐seq profiling reveals different pathways between remodeled vessels and myocardium in hypertrophic cardiomyopathy
Source: Microcirculation. 2022 Oct 14;29(8):e12790. doi: 10.1111/micc.12790 (PMC9787970; doi:10.1111/micc.12790)
Supplement: Supplementary file 5 — Figures S1–S12 [file MICC-29-e12790-s004.pdf]

RNA-seq profiling reveals different pathways between remodelled vessels and myocardium in Hypertrophic Cardiomyopathy

Annalinda Pisano[1] PhD, Loredana Le Pera[2] PhD, Raffaella Carletti[1] LT, Bruna Cerbelli[1] MD PhD, Maria Gemma Pignataro[1] PhD, Angelina Pernazza[1] MD, Fabrizio Ferre[3] PhD, Maria Lombardi[4] Msc, Davide Lazzeroni[4] MD, Iacopo Olivotto[5] MD, Ornella E Rimoldi[6] MD, Chiara Foglieni[4] PhD, Paolo G Camici[4, 7] MD, Giulia d'Amati[1]* MD PhD

*Corresponding author: Giulia d'Amati, Department of Radiological, Oncological and Pathological Sciences, Sapienza University of Rome and Policlinico Umberto I, Viale Regina Elena 324, 00161 Rome, Italy. giulia.damati@uniroma1.it Tel. +390649973332 Fax. +39064461484

Supplementary Figure S1. LCM procedure

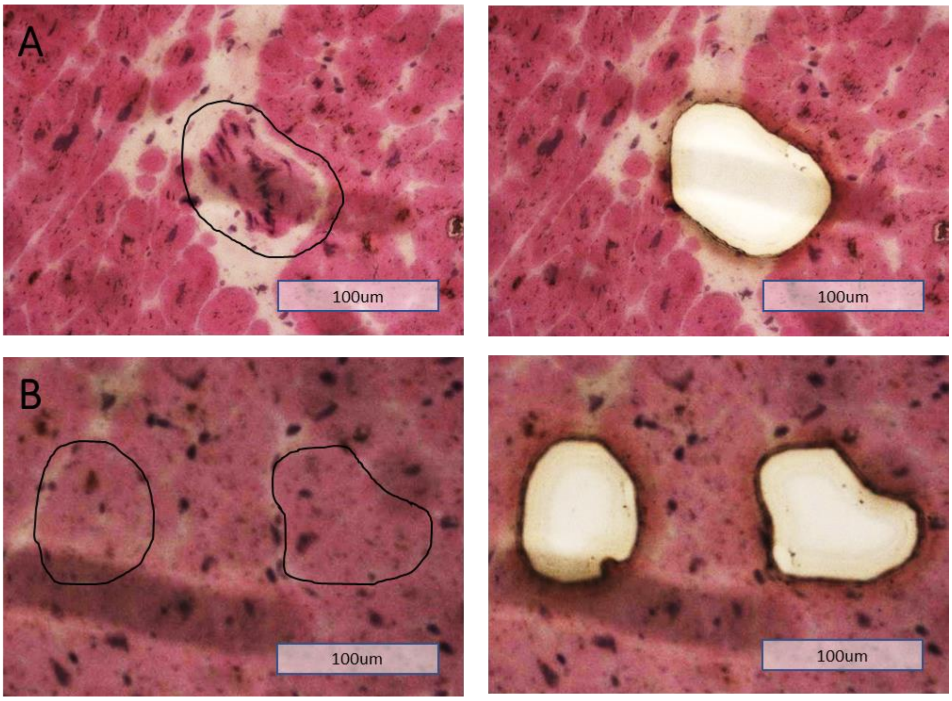

Supplementary Figure S2. Morphologic features of HCM myectomy samples

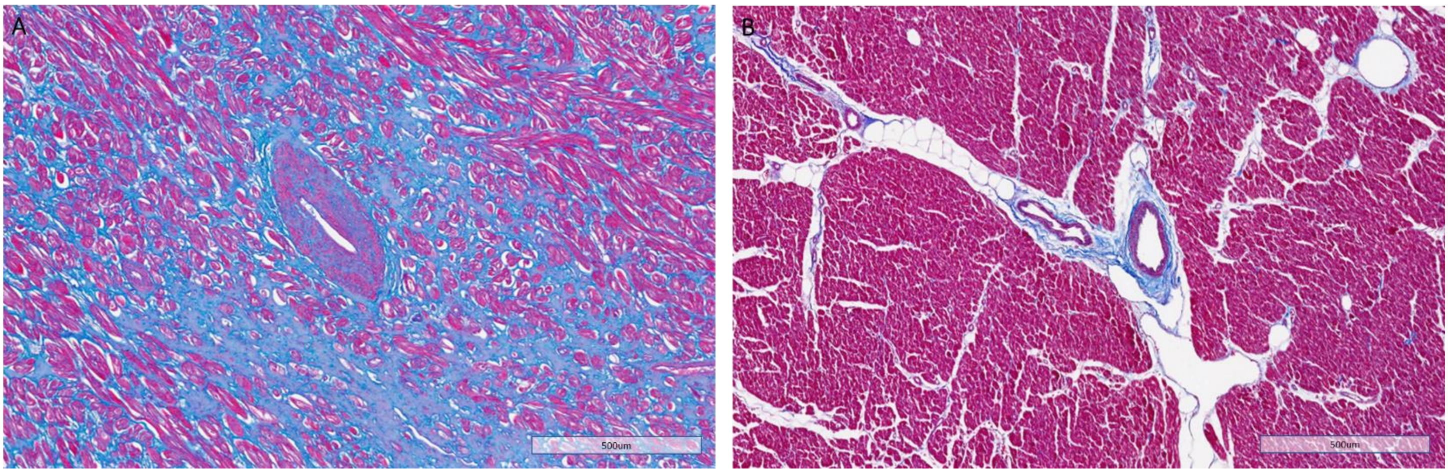

Supplementary Figure S3. Read quality filtering and mapping (cardiomyocytes)

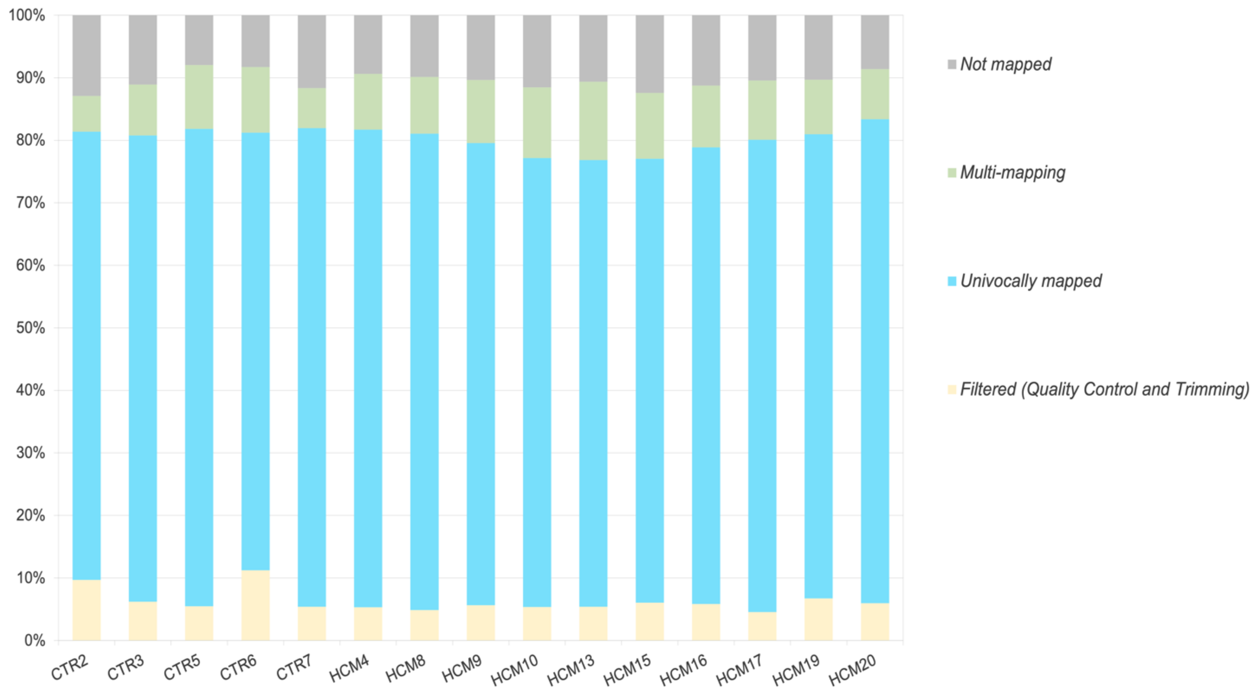

Supplementary Figure S4. Read quality filtering and mapping (arterioles)

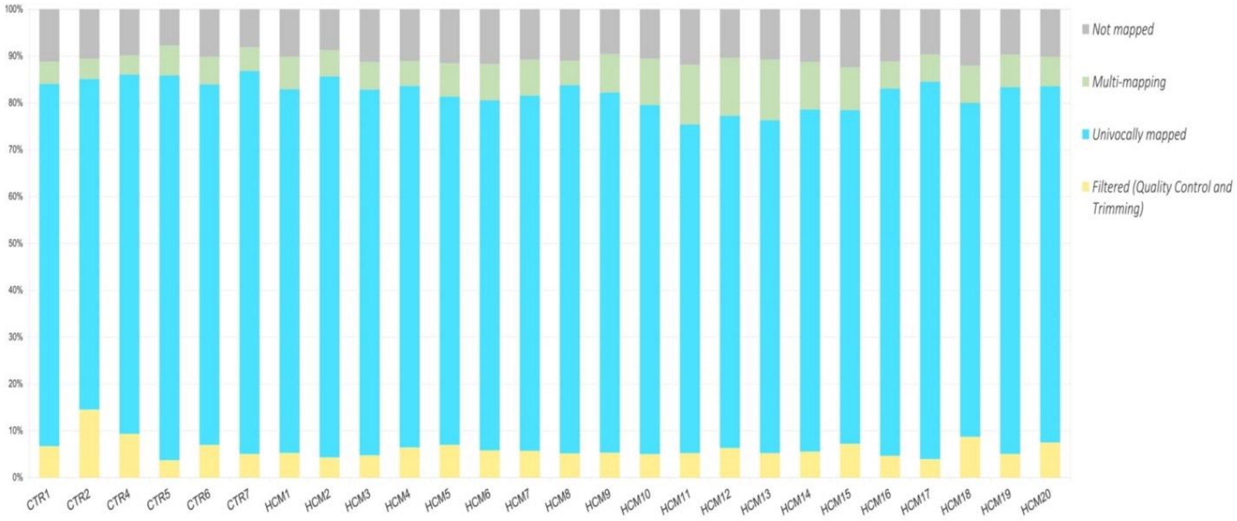

Supplementary Figure S5. PCA (cardiomyocytes)

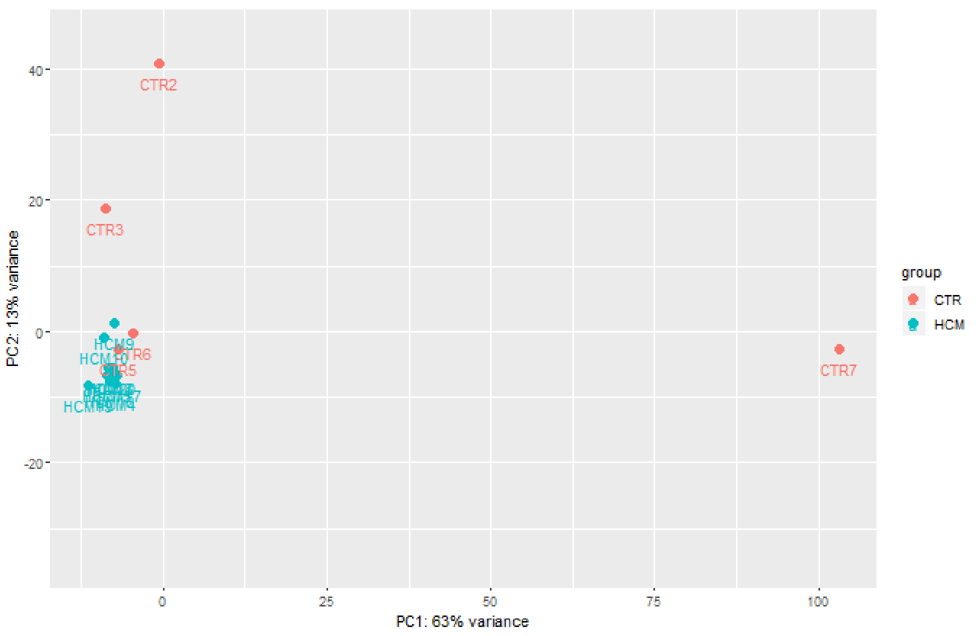

Supplementary Figure S6. PCA (arterioles)

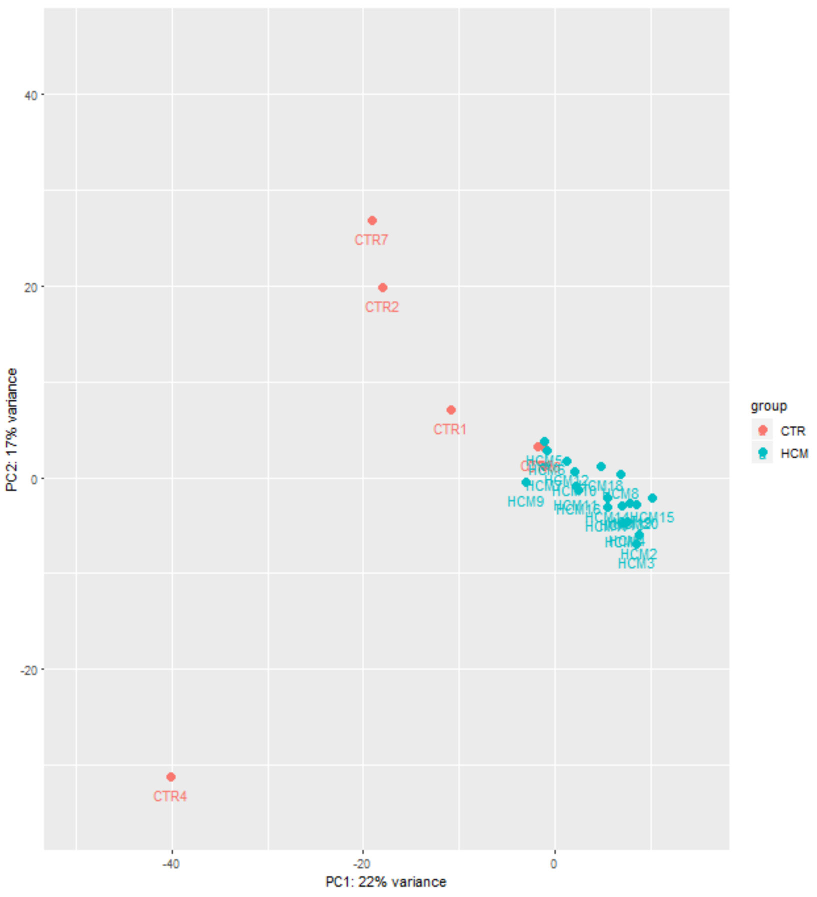

Supplementary Figure S7. Comparison of GO term enrichment analysis for all DEGs

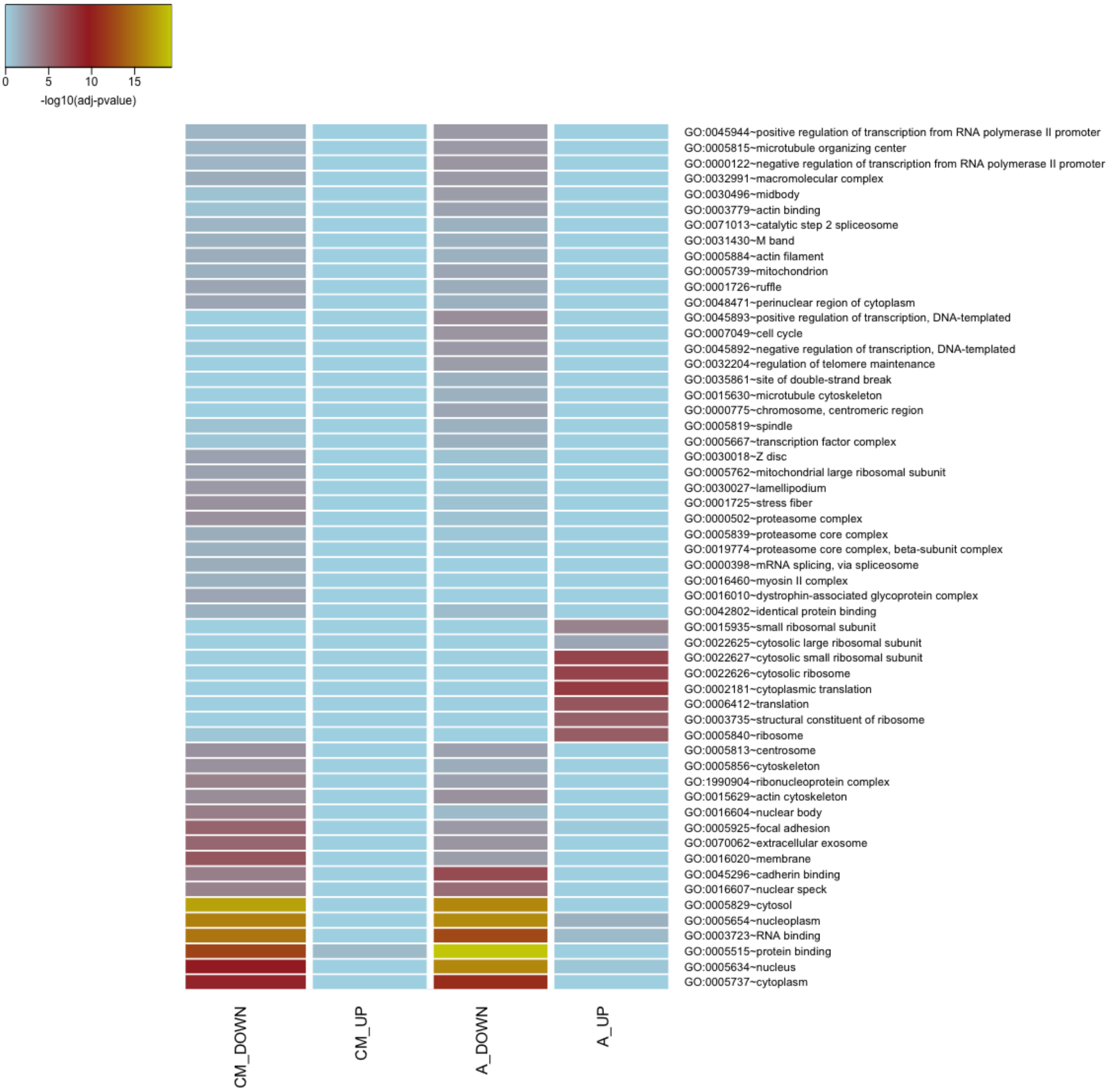

Supplementary Figure S8. Comparison of Pathways and InterPro enrichment analysis for all DEGs

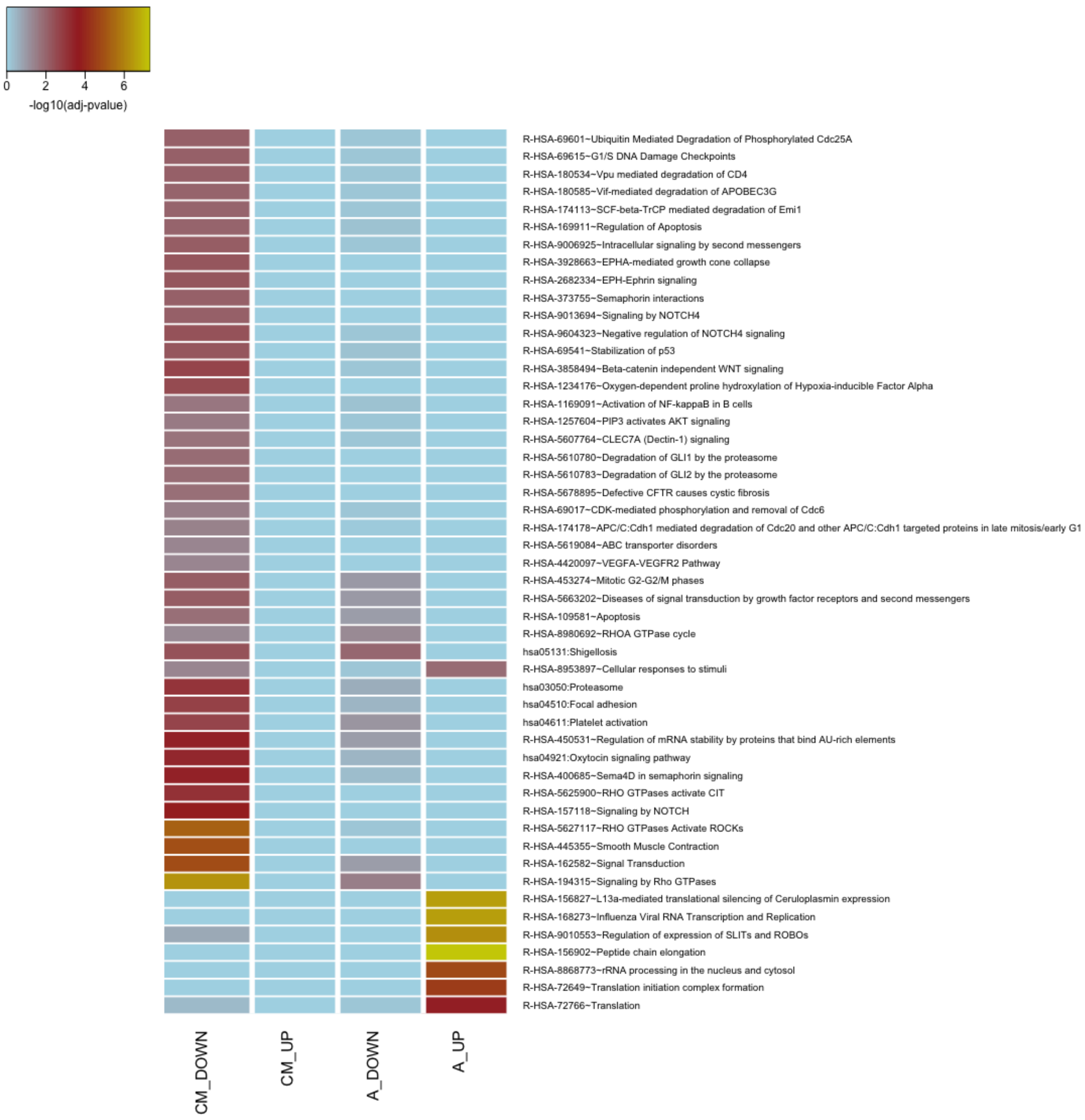

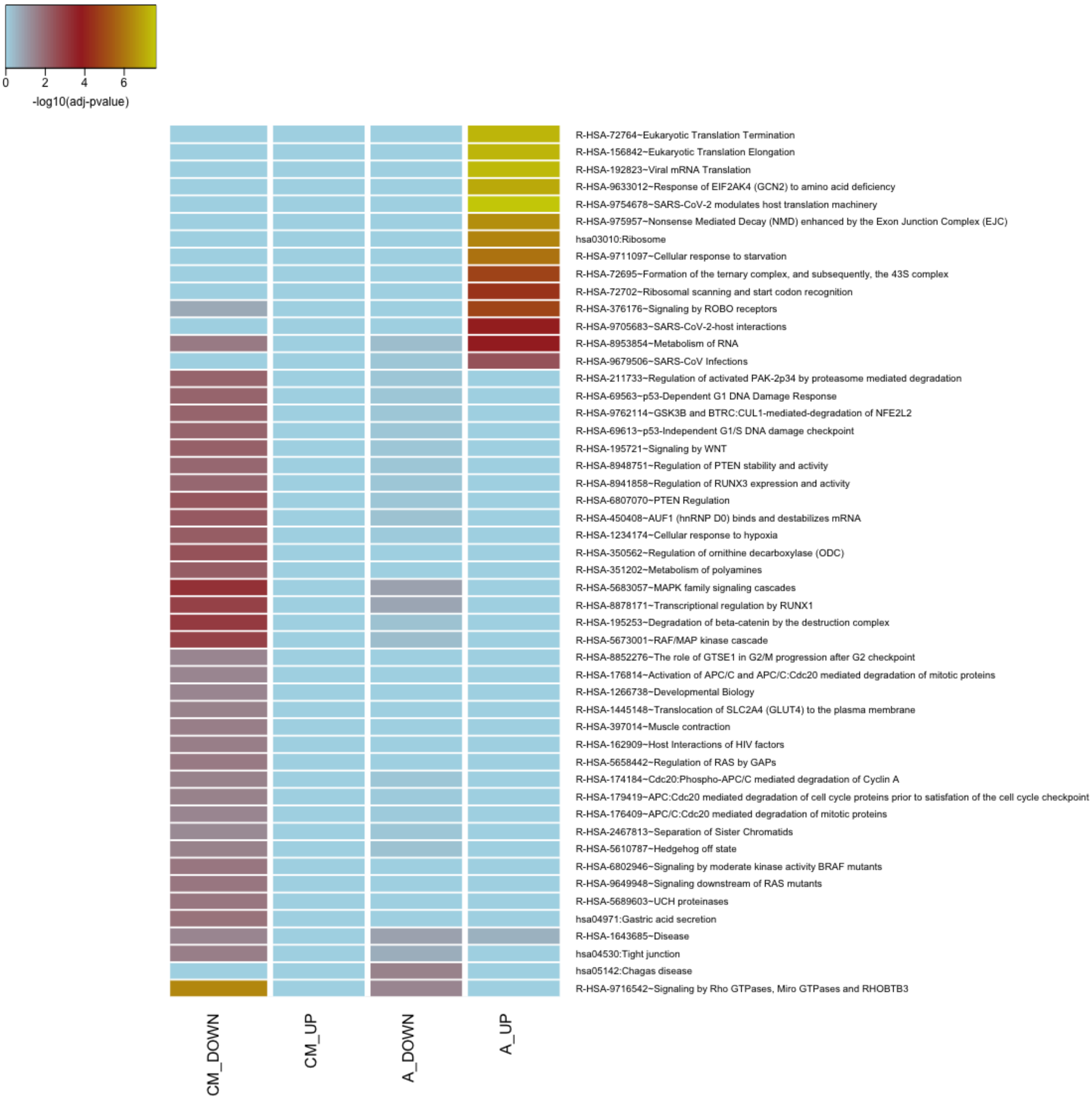

0
2
4
6
-log10(adj-pvalue)
R-HSA-72764~Eukaryotic Translation Termination
R-HSA-156842~Eukaryotic Translation Elongation
R-HSA-192823~Viral mRNA Translation
R-HSA-9633012~Response of EIF2AK4 (GCN2) to amino acid deficiency
R-HSA-9754678~SARS-CoV-2 modulates host translation machinery
R-HSA-975957~Nonsense Mediated Decay (NMD) enhanced by the Exon Junction Complex (EJC)
hsa03010:Ribosome
R-HSA-9711097~Cellular response to starvation
R-HSA-72695~Formation of the ternary complex, and subsequently, the 43S complex
R-HSA-72702~Ribosomal scanning and start codon recognition
R-HSA-376176~Signaling by ROBO receptors
R-HSA-9705683~SARS-CoV-2-host interactions
R-HSA-8953854~Metabolism of RNA
R-HSA-9679506~SARS-CoV Infections
R-HSA-211733~Regulation of activated PAK-2p34 by proteasome mediated degradation
R-HSA-69563~p53-Dependent G1 DNA Damage Response
R-HSA-9762114~GSK3B and BTRC:CUL1-mediated-degradation of NFE2L2
R-HSA-69613~p53-Independent G1/S DNA damage checkpoint
R-HSA-195721~Signaling by WNT
R-HSA-8948751~Regulation of PTEN stability and activity
R-HSA-8941858~Regulation of RUNX3 expression and activity
R-HSA-6807070~PTEN Regulation
R-HSA-450408~AUF1 (hnRNP D0) binds and destabilizes mRNA
R-HSA-1234174~Cellular response to hypoxia
R-HSA-350562~Regulation of ornithine decarboxylase (ODC)
R-HSA-351202~Metabolism of polyamines
R-HSA-5683057~MAPK family signaling cascades
R-HSA-8878171~Transcriptional regulation by RUNX1
R-HSA-195253~Degradation of beta-catenin by the destruction complex
R-HSA-5673001~RAF/MAP kinase cascade
R-HSA-8852276~The role of GTSE1 in G2/M progression after G2 checkpoint
R-HSA-176814~Activation of APC/C and APC/C:Cdc20 mediated degradation of mitotic proteins
R-HSA-1266738~Developmental Biology
R-HSA-1445148~Translocation of SLC2A4 (GLUT4) to the plasma membrane
R-HSA-397014~Muscle contraction
R-HSA-162909~Host Interactions of HIV factors
R-HSA-5658442~Regulation of RAS by GAPs
R-HSA-174184~Cdc20:Phospho-APC/C mediated degradation of Cyclin A
R-HSA-179419~APC:Cdc20 mediated degradation of cell cycle proteins prior to satisfation of the cell cycle checkpoint
R-HSA-176409~APC/C:Cdc20 mediated degradation of mitotic proteins
R-HSA-2467813~Separation of Sister Chromatids
R-HSA-5610787~Hedgehog off state
R-HSA-6802946~Signaling by moderate kinase activity BRAF mutants
R-HSA-9649948~Signaling downstream of RAS mutants
R-HSA-5689603~UCH proteinases
hsa04971:Gastric acid secretion
R-HSA-1643685~Disease
hsa04530:Tight junction
hsa05142:Chagas disease
R-HSA-9716542~Signaling by Rho GTPases, Miro GTPases and RHOBTB3
CM_DOWN
CM_UP
A_DOWN
A_UP

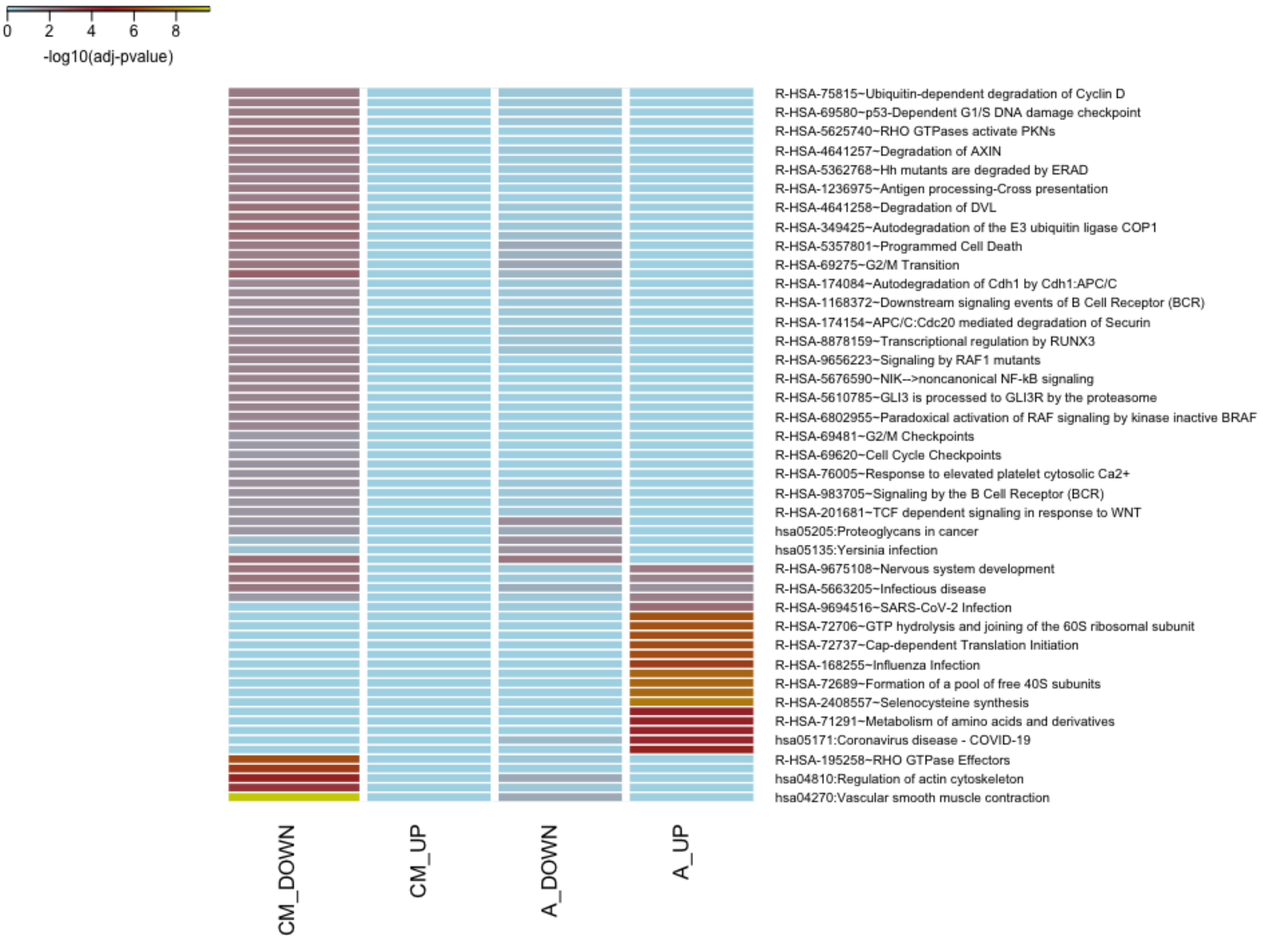

0 2 4 6 8
-log10(adj-pvalue)
R-HSA-75815~Ubiquitin-dependent degradation of Cyclin D
R-HSA-69580~p53-Dependent G1/S DNA damage checkpoint
R-HSA-5625740~RHO GTPases activate PKNs
R-HSA-4641257~Degradation of AXIN
R-HSA-5362768~Hh mutants are degraded by ERAD
R-HSA-1236975~Antigen processing-Cross presentation
R-HSA-4641258~Degradation of DVL
R-HSA-349425~Autodegradation of the E3 ubiquitin ligase COP1
R-HSA-5357801~Programmed Cell Death
R-HSA-69275~G2/M Transition
R-HSA-174084~Autodegradation of Cdh1 by Cdh1:APC/C
R-HSA-1168372~Downstream signaling events of B Cell Receptor (BCR)
R-HSA-174154~APC/C:Cdc20 mediated degradation of Securin
R-HSA-8878159~Transcriptional regulation by RUNX3
R-HSA-9656223~Signaling by RAF1 mutants
R-HSA-5676590~NIK-->noncanonical NF-kB signaling
R-HSA-5610785~GLI3 is processed to GLI3R by the proteasome
R-HSA-6802955~Paradoxical activation of RAF signaling by kinase inactive BRAF
R-HSA-69481~G2/M Checkpoints
R-HSA-69620~Cell Cycle Checkpoints
R-HSA-76005~Response to elevated platelet cytosolic Ca2+
R-HSA-983705~Signaling by the B Cell Receptor (BCR)
R-HSA-201681~TCF dependent signaling in response to WNT
hsa05205:Proteoglycans in cancer
hsa05135:Yersinia infection
R-HSA-9675108~Nervous system development
R-HSA-5663205~Infectious disease
R-HSA-9694516~SARS-CoV-2 Infection
R-HSA-72706~GTP hydrolysis and joining of the 60S ribosomal subunit
R-HSA-72737~Cap-dependent Translation Initiation
R-HSA-168255~Influenza Infection
R-HSA-72689~Formation of a pool of free 40S subunits
R-HSA-2408557~Selenocysteine synthesis
R-HSA-71291~Metabolism of amino acids and derivatives
hsa05171:Coronavirus disease - COVID-19
R-HSA-195258~RHO GTPase Effectors
hsa04810:Regulation of actin cytoskeleton
hsa04270:Vascular smooth muscle contraction
CM_DOWN
CM_UP
A_DOWN
A_UP

Supplementary Figure S9. Comparison of UniProt annotation enrichment analysis for all DEGs

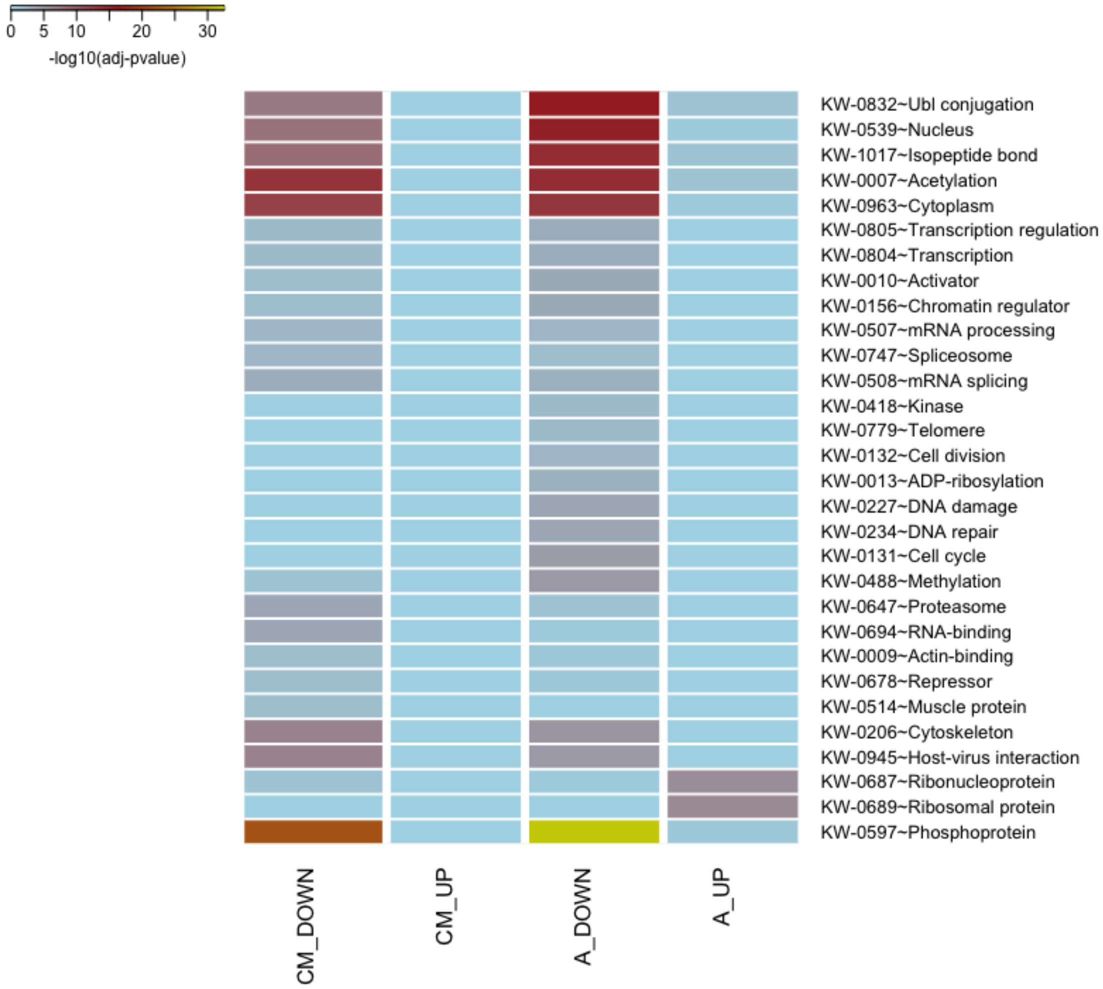

Supplementary Figure S10. Comparison of IntAct annotation enrichment analysis for all DEGs

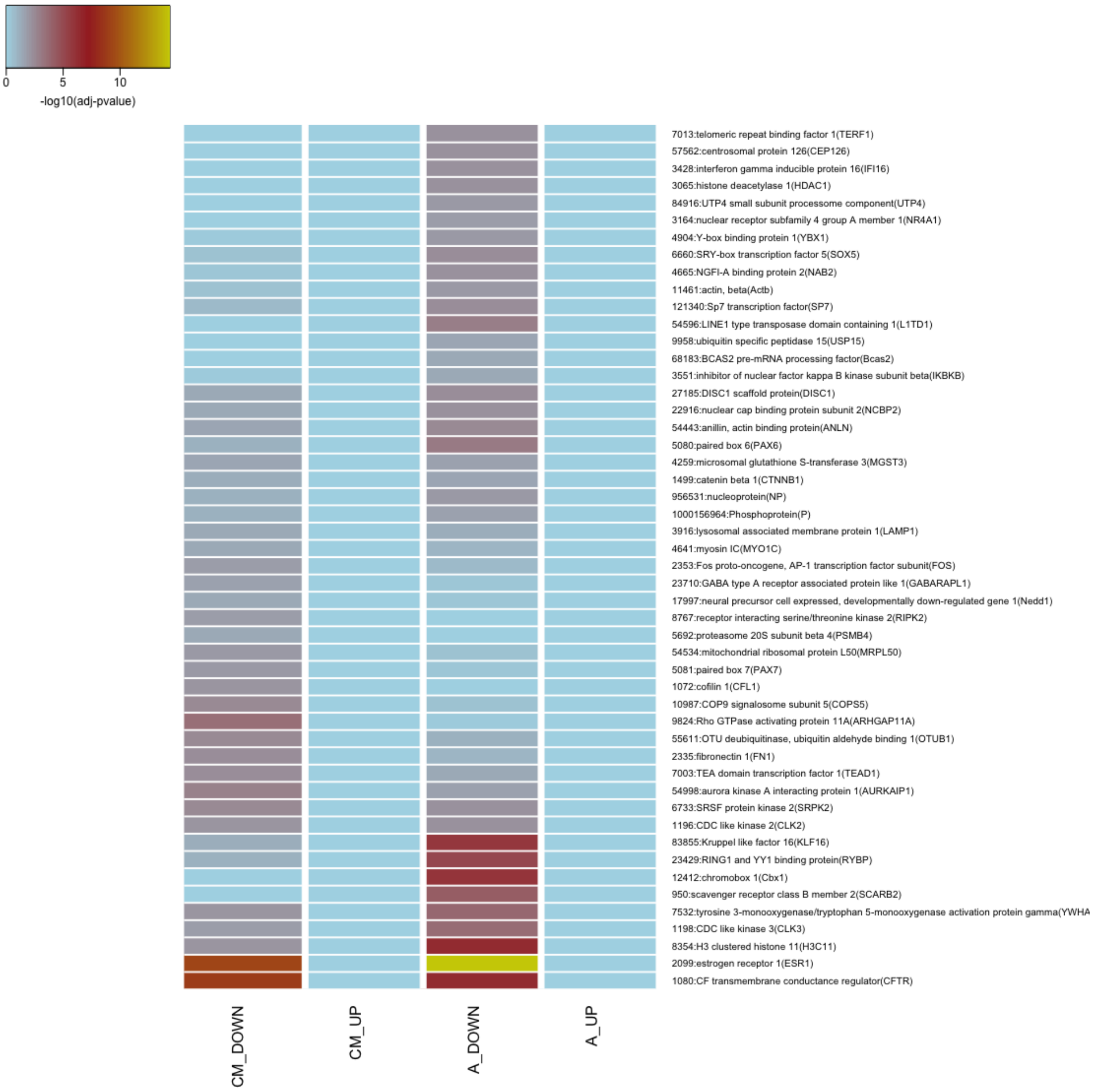

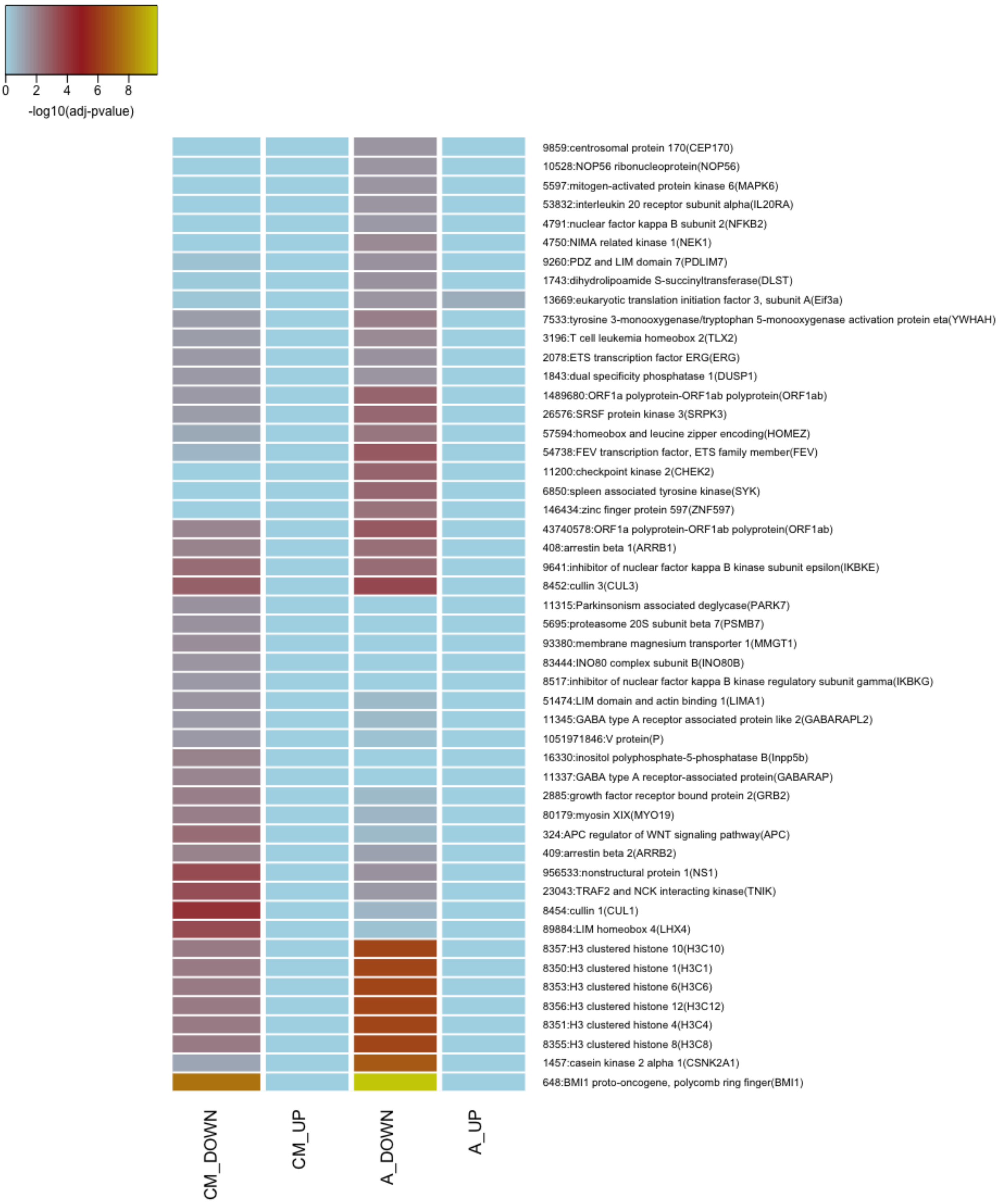
0
2
4
6
8
-log10(adj-pvalue)
9859:centrosomal protein 170(CEP170)
10528:NOP56 ribonucleoprotein(NOP56)
5597:mitogen-activated protein kinase 6(MAPK6)
53832:interleukin 20 receptor subunit alpha(IL20RA)
4791:nuclear factor kappa B subunit 2(NFKB2)
4750:NIMA related kinase 1(NEK1)
9260:PDZ and LIM domain 7(PDLIM7)
1743:dihydrolipoamide S-succinyltransferase(DLST)
13669:eukaryotic translation initiation factor 3, subunit A(Eif3a)
7533:tyrosine 3-monooxygenase/tryptophan 5-monooxygenase activation protein eta(YWHAH)
3196:T cell leukemia homeobox 2(TLX2)
2078:ETS transcription factor ERG(ERG)
1843:dual specificity phosphatase 1(DUSP1)
1489680:ORF1a polyprotein-ORF1ab polyprotein(ORF1ab)
26576:SRSF protein kinase 3(SRPK3)
57594:homeobox and leucine zipper encoding(HOMEZ)
54738:FEV transcription factor, ETS family member(FEV)
11200:checkpoint kinase 2(CHEK2)
6850:spleen associated tyrosine kinase(SYK)
146434:zinc finger protein 597(ZNF597)
43740578:ORF1a polyprotein-ORF1ab polyprotein(ORF1ab)
408:arrestin beta 1(ARRB1)
9641:inhibitor of nuclear factor kappa B kinase subunit epsilon(IKBKE)
8452:cullin 3(CUL3)
11315:Parkinsonism associated deglycase(PARK7)
5695:proteasome 20S subunit beta 7(PSMB7)
93380:membrane magnesium transporter 1(MMGT1)
83444:INO80 complex subunit B(INO80B)
8517:inhibitor of nuclear factor kappa B kinase regulatory subunit gamma(IKBKG)
51474:LIM domain and actin binding 1(LIMA1)
11345:GABA type A receptor associated protein like 2(GABARAPL2)
1051971846:V protein(P)
16330:inositol polyphosphate-5-phosphatase B(Inpp5b)
11337:GABA type A receptor-associated protein(GABARAP)
2885:growth factor receptor bound protein 2(GRB2)
80179:myosin XIX(MYO19)
324:APC regulator of WNT signaling pathway(APC)
409:arrestin beta 2(ARRB2)
956533:nonstructural protein 1(NS1)
23043:TRAF2 and NCK interacting kinase(TNIK)
8454:cullin 1(CUL1)
89884:LIM homeobox 4(LHX4)
8357:H3 clustered histone 10(H3C10)
8350:H3 clustered histone 1(H3C1)
8353:H3 clustered histone 6(H3C6)
8356:H3 clustered histone 12(H3C12)
8351:H3 clustered histone 4(H3C4)
8355:H3 clustered histone 8(H3C8)
1457:casein kinase 2 alpha 1(CSNK2A1)
648:BMI1 proto-oncogene, polycomb ring finger(BMI1)
CM_DOWN
CM_UP
A_DOWN
A_UP

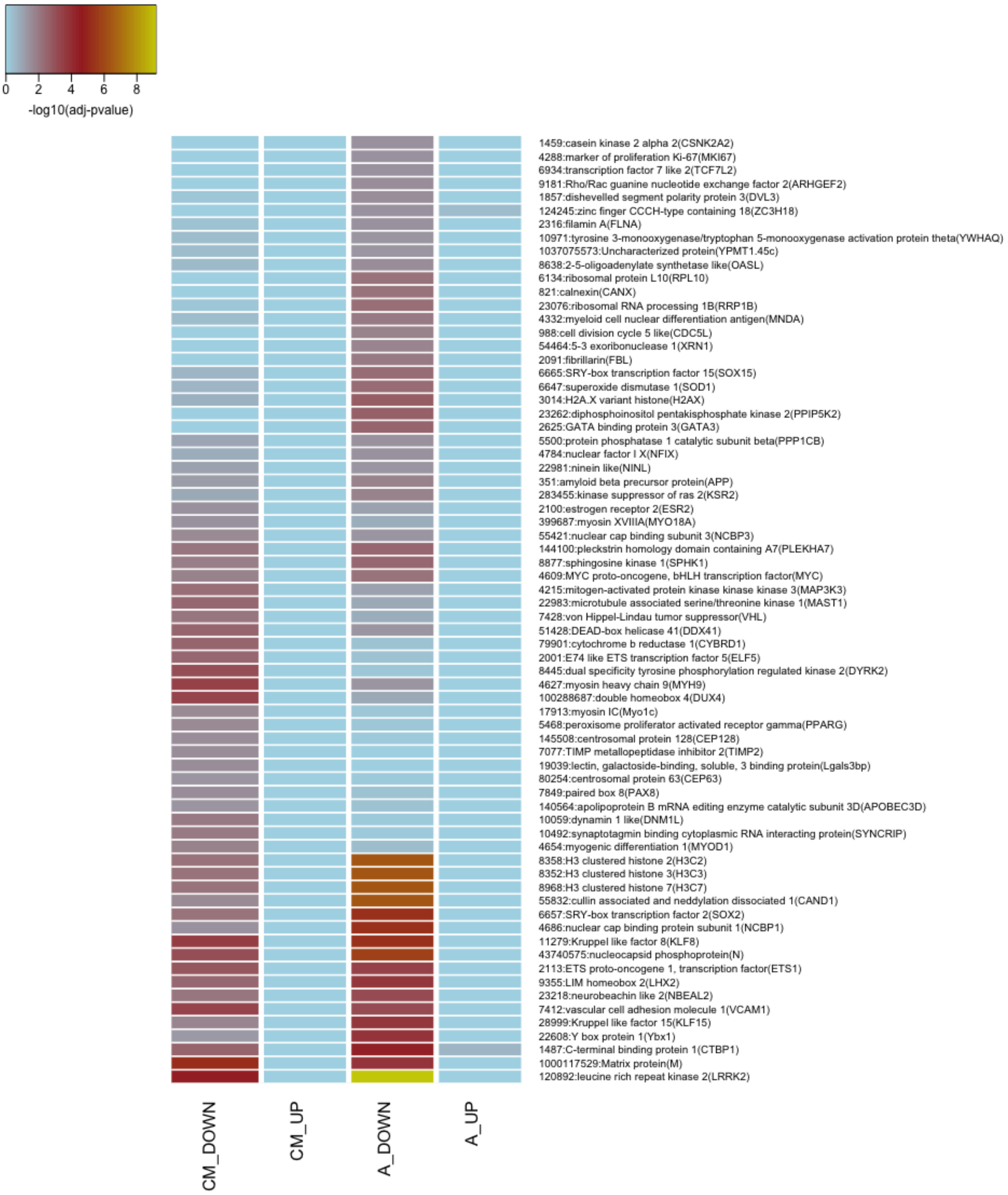
0
2
4
6
8
-log10(adj-pvalue)
1459:casein kinase 2 alpha 2(CSNK2A2)
4288:marker of proliferation Ki-67(MKI67)
6934:transcription factor 7 like 2(TCF7L2)
9181:Rho/Rac guanine nucleotide exchange factor 2(ARHGEF2)
1857:dishevelled segment polarity protein 3(DVL3)
124245:zinc finger CCCH-type containing 18(ZC3H18)
2316:filamin A(FLNA)
10971:tyrosine 3-monooxygenase/tryptophan 5-monooxygenase activation protein theta(YWHAQ)
1037075573:Uncharacterized protein(YPMT1.45c)
8638:2-5-oligoadenylate synthetase like(OASL)
6134:ribosomal protein L10(RPL10)
821:calnexin(CANX)
23076:ribosomal RNA processing 1B(RRP1B)
4332:myeloid cell nuclear differentiation antigen(MNDA)
988:cell division cycle 5 like(CDC5L)
54464:5-3 exoribonuclease 1(XRN1)
2091:fibrillarin(FBL)
6665:SRY-box transcription factor 15(SOX15)
6647:superoxide dismutase 1(SOD1)
3014:H2A.X variant histone(H2AX)
23262:diphosphoinositol pentakisphosphate kinase 2(PPIP5K2)
2625:GATA binding protein 3(GATA3)
5500:protein phosphatase 1 catalytic subunit beta(PPP1CB)
4784:nuclear factor I X(NFIX)
22981:ninein like(NINL)
351:amyloid beta precursor protein(APP)
283455:kinase suppressor of ras 2(KSR2)
2100:estrogen receptor 2(ESR2)
399687:myosin XVIIIA(MYO18A)
55421:nuclear cap binding subunit 3(NCBP3)
144100:pleckstrin homology domain containing A7(PLEKHA7)
8877:sphingosine kinase 1(SPHK1)
4609:MYC proto-oncogene, bHLH transcription factor(MYC)
4215:mitogen-activated protein kinase kinase kinase 3(MAP3K3)
22983:microtubule associated serine/threonine kinase 1(MAST1)
7428:von Hippel-Lindau tumor suppressor(VHL)
51428:DEAD-box helicase 41(DDX41)
79901:cytochrome b reductase 1(CYBRD1)
2001:E74 like ETS transcription factor 5(ELF5)
8445:dual specificity tyrosine phosphorylation regulated kinase 2(DYRK2)
4627:myosin heavy chain 9(MYH9)
100288687:double homeobox 4(DUX4)
17913:myosin IC(Myo1c)
5468:peroxisome proliferator activated receptor gamma(PPARG)
145508:centrosomal protein 128(CEP128)
7077:TIMP metallopeptidase inhibitor 2(TIMP2)
19039:lectin, galactoside-binding, soluble, 3 binding protein(Lgals3bp)
80254:centrosomal protein 63(CEP63)
7849:paired box 8(PAX8)
140564:apolipoprotein B mRNA editing enzyme catalytic subunit 3D(APOBEC3D)
10059:dynamin 1 like(DNM1L)
10492:synaptotagmin binding cytoplasmic RNA interacting protein(SYNCRIP)
4654:myogenic differentiation 1(MYOD1)
8358:H3 clustered histone 2(H3C2)
8352:H3 clustered histone 3(H3C3)
8968:H3 clustered histone 7(H3C7)
55832:cullin associated and neddylation dissociated 1(CAND1)
6657:SRY-box transcription factor 2(SOX2)
4686:nuclear cap binding protein subunit 1(NCBP1)
11279:Kruppel like factor 8(KLF8)
43740575:nucleocapsid phosphoprotein(N)
2113:ETS proto-oncogene 1, transcription factor(ETS1)
9355:LIM homeobox 2(LHX2)
23218:neurobeachin like 2(NBEAL2)
7412:vascular cell adhesion molecule 1(VCAM1)
28999:Kruppel like factor 15(KLF15)
22608:Y box protein 1(Ybx1)
1487:C-terminal binding protein 1(CTBP1)
1000117529:Matrix protein(M)
120892:leucine rich repeat kinase 2(LRRK2)
CM_DOWN
CM_UP
A_DOWN
A_UP

Supplementary Figure S11. Comparison of GO term enrichment analysis for exclusive DEGs

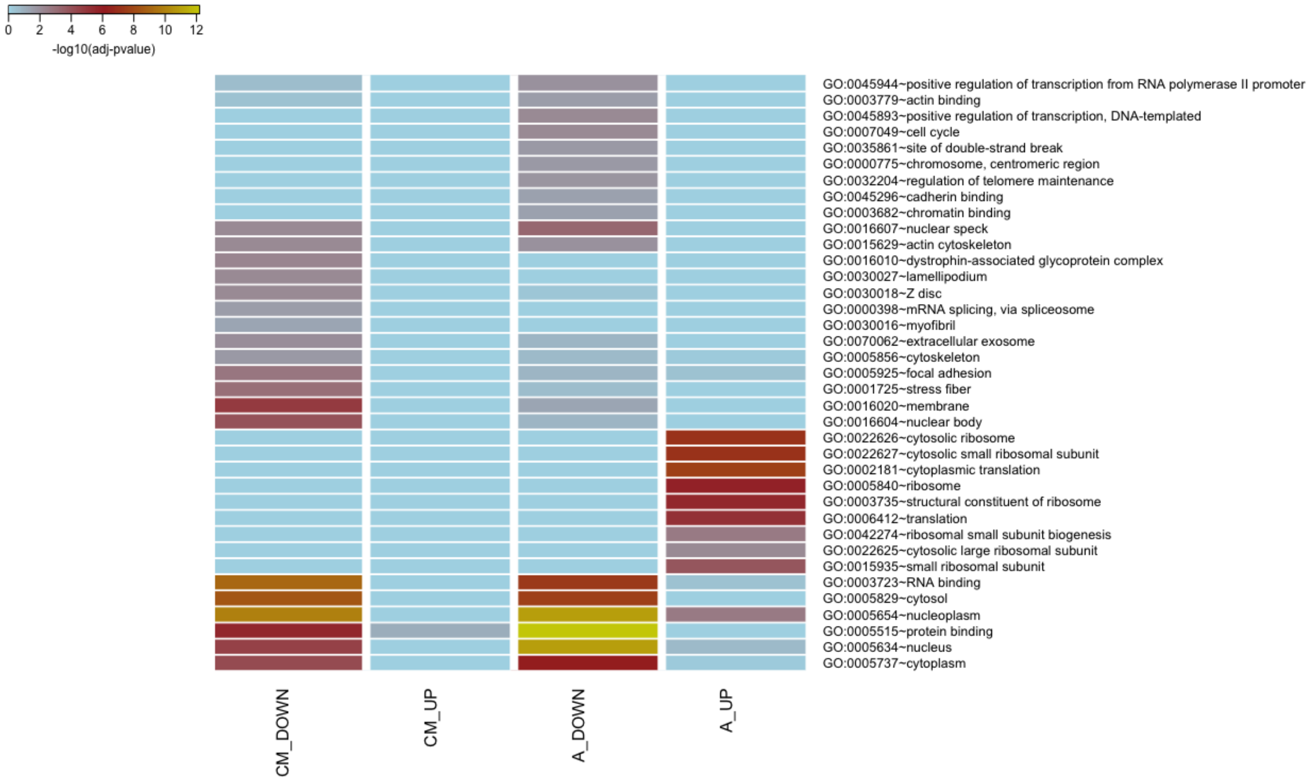

Supplementary Figure S12. Comparison of UniProt annotation enrichment analysis for exclusive DEGs

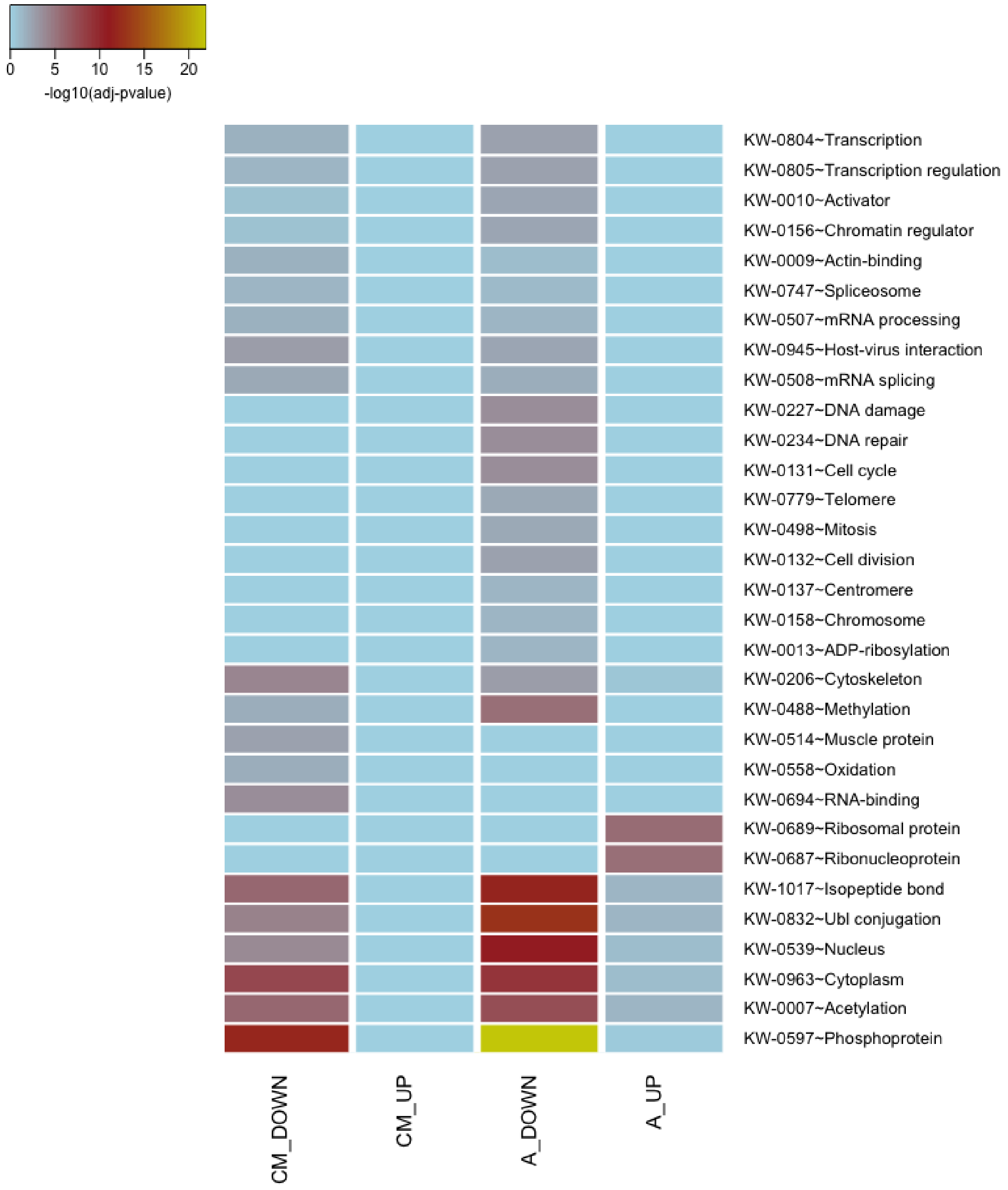

Supplementary Figure caption:

Supplementary Figure S1

Representative image of myectomy samples before and after microdissection of arterioles (**A**) or single cardiomyocytes (**B**) (Eematoxylin and Eosin stain).

Supplementary Figure S2

Azan-Mallory stain shows myofiber disarray, interstitial fibrosis and remodelled coronary arterioles in HCM samples (**A**). Control samples show a normal myocardial and vessel structure (**B**).

Supplementary Figures S3-4

Histograms of reads aligned to the human reference genome (GRCh38) are reported for each sample of cardiomyocytes (**Fig. S3**) and arterioles (**Fig. S4**). In yellow, the reads discarded after pre-processing analysis performed by using FastQC and Trimmomatic tools; in cyan, the reads univocally aligned to the human reference genome by using HISAT2 aligner; in light green, the reads with more than one match on the reference genome; in grey, the reads not aligned. Only univocally mapped reads in each sample (about 70% of the sequenced reads) were kept for gene expression estimation**.**

Supplementary Figures S5-6

Principal Component Analysis (PCA) of HCM and CTRL gene expression data in cardiomyocytes (**Fig. S5**) and arterioles (**Fig. S6**) samples. The first two principal components are plotted, identifying (even if not clearly separated) two clusters of samples corresponding to HCM (in red) and CTRL (in cyan) data. Percentage of variation explained by each component are indicated along the axes.

Supplementary Figures S7-10

Comparison of different collections of functional biological categories enriched in the list of DEGs. All terms statistically enriched in at least one of the four groups of DEGs, cardiomyocytes_DOWN (CM_DOWN), cardiomyocytes_UP (CM_UP), arterioles_DOWN (A_DOWN), arterioles_UP (A_UP), are reported in the heatmaps, with adjusted p-values plotted in blue-yellow scale color, where yellow indicates higher significant results. In the figures are shown the heatmaps related to enriched GO terms (**Fig. S7**), enriched KEGG and Reactome Pathways and InterPro families (**Fig. S8**), enriched Uniprot annotation terms (**Fig. S9**), enriched IntAct categories (**Fig. S10**).

Supplementary Figures S11-12

Comparison of different biological functional categories enriched in the list of “exclusive DEGs”. All terms statistically enriched in at least one of the four groups of “exclusive DEGs”, cardiomyocytes_DOWN (CM_DOWN), cardiomyocytes_UP (CM_UP), arterioles_DOWN (A_DOWN), arterioles_UP (A_UP), are reported in the heatmaps, with adjusted p-values plotted in blue-yellow scale color, where yellow indicates higher significant results. In the figures are shown the heatmaps related to enriched GO terms (**Fig. S11**), and enriched Uniprot annotation terms (**Fig. S12**).
